# Extracellular vesicles contribute to the beneficial effects of exercise training in APP/PS1 mice

**DOI:** 10.1016/j.isci.2025.111752

**Published:** 2025-01-04

**Authors:** Oliver K. Fuller, Emma D. McLennan, Casey L. Egan, Nimna Perera, Lauren V. Terry, Jae Pyun, Mariana de Mendonca, Guilherme Defante Telles, Benoit Smeuninx, Emma L. Burrows, Ghizal Siddiqui, Darren J. Creek, John W. Scott, Michael A. Pearen, Pamali Fonseka, Joseph A. Nicolazzo, Suresh Mathivanan, Anthony J. Hannan, Grant A. Ramm, Martin Whitham, Mark A. Febbraio

**Affiliations:** 1 Monash Institute of Pharmaceutical Sciences, Monash University, Melbourne, VIC, Australia; 2 Florey Institute of Neuroscience and Mental Health, University of Melbourne, Melbourne, VIC, Australia; 3 QIMR Berghofer Medical Research Institute, Brisbane, QLD, Australia; 4 La Trobe Institute for Molecular Science, La Trobe University, Melbourne, VIC, Australia; 5 School of Sport, Exercise & Rehabilitation Sciences, University of Birmingham, Edgbaston, Birmingham, UK

**Keywords:** Molecular physiology, Neuroscience, Cell biology

## Abstract

Exercise improves cognitive function in Alzheimer’s disease (AD) via mechanism that are not fully clear. Here, we first examined the effect of voluntary exercise training (VET) on energy metabolism and cognitive function in the APP/PS1 transgenic mouse (Tg) model of familial AD. Next, we profiled extracellular vesicles (EVs) and examined whether they may play a role in the protective effects of VET via intranasal administration of EVs, purified from the blood of sedentary (sEV) and/or acutely exercised (eEV) donor wild-type mice into APP/PS1Tg mice. We show that VET reduced resting energy expenditure (REE) and improved cognition in APP/PS1 Tg mice. Administration of eEV, but not sEV, also reduced REE, but had no effect on cognition. Taken together, these data show that exercise is effective intervention to improve symptoms of AD in APP/PS1Tg mice. In addition, eEVs mediate some of these effects, implicating EVs in the treatment of age-related neurodegenerative diseases.

## Highlights

• Exercise restores aberrant resting energy expenditure in APP/PS1 mice
• Exercise improves memory and reduces cortical Aβ in APP/PS1 mice
• Exercise EVs (eEVs) differ in miRNA and protein cargo, impacting energy metabolism
• EVs reduce energy expenditure in APP/PS1 mice but do not improve memory or Aβ

## Graphical abstract

**Figure:**
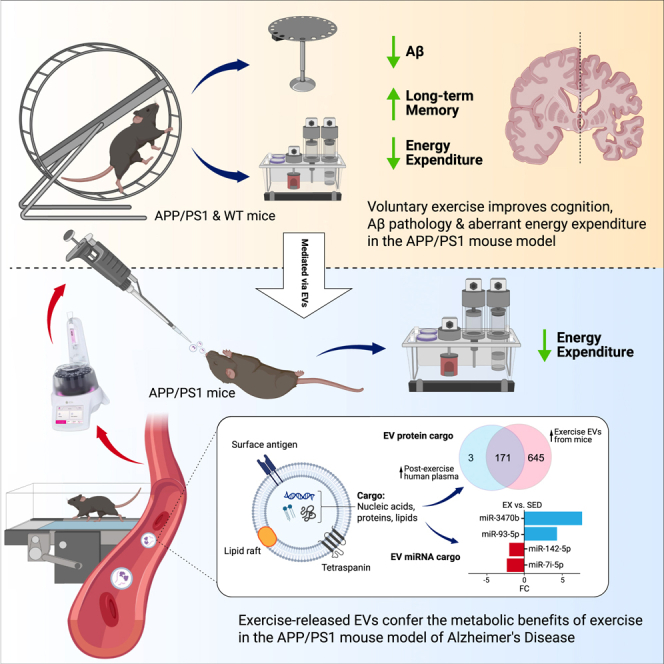

## Introduction

The number of people aged >60 years is expected to grow from 0.9 to more than 1.4 billion by 2030,^1^ increasing the incidence of age-related neurodegenerative diseases, such as Alzheimer’s disease (AD), for which there are currently few, if any, pharmacological treatment options. AD is characterized by progressive cognitive decline associated with the presence of amyloid-beta (Aβ) accumulation. Current estimates suggest that the global economic burden of dementia is >$800 billion, with significant increases expected over the next 10 years.^2^ Therefore, interventions that prevent or delay the progression of AD have the potential to significantly impact the quality of life of older individuals. Changes in metabolism, including weight loss and increased energy expenditure, are often observed in individuals with AD^3^ independent of alterations in energy intake.^4^^,^^5^^,^^6^ Several studies have also shown that individuals with AD have increased resting energy expenditure (REE) compared with age-matched controls.^7^^,^^8^^,^^9^ While it was thought that changes in total energy expenditure (TEE) would also occur, potentially due to an increase in physical activity from wandering, individuals with AD are less physically active and display a reduction in intensity when undertaking tasks such as walking.^10^^,^^11^ Studies in transgenic mouse models of AD have supported these results.^12^^,^^13^^,^^14^ The importance of both peripheral and brain-specific metabolic function and their relationship with brain diseases, such as AD, have been known for some time. People with type 2 diabetes mellitus (T2DM), for example, have double the risk of developing AD.^15^^,^^16^ Very little, however, is known regarding the mechanism/s responsible for these observations. This growing understanding of metabolic dysfunction’s role in AD underscores the need for exploring innovative interventions that target these pathways, such as the potential benefits of physical activity and exercise in modulating metabolic and cognitive functions.

Physical activity and fitness levels are associated with the maintenance or improvement of brain biology and function, slowing progression and potentially preventing brain diseases such as AD.^17^^,^^18^^,^^19^ The exact mechanisms underlying these beneficial effects are poorly understood but seem to be driven by several factors, including enhanced neurogenesis,^20^^,^^21^^,^^22^ decreased Aβ pathology through improved clearance,^21^ and modulation of neuroinflammation.^23^ Physical activity may also modulate metabolic regulation within the brain and peripheral systems.^24^ As metabolic dysfunction becomes increasingly appreciated in driving disease progression in AD, understanding how exercise targets these pathways may provide a greater understanding of the fundamental biological processes involved, potentially leading to the development of effective therapies.^25^ Largely driven by the complex perturbation of homeostasis that occurs in all tissues and subsequent adaptations that take place, the metabolic benefits of physical activity appear to go beyond the simple management of energy balance and body weight.^26^^,^^27^^,^^28^ After acute exercise in healthy humans, whole-body insulin sensitivity improves along with increased glucose uptake in skeletal muscle, thereby increasing glucose tolerance.^29^^,^^30^^,^^31^ These effects on glucose metabolism are replicated in mouse models of AD, along with decreases in white adipose tissue, triglycerides, and low-density lipoprotein (LDL) cholesterol, and increases in high-density lipoprotein (HDL) cholesterol^32^^,^^33^^,^^34^^,^^35^ Along with these improvements in energy metabolism, improvements in cognitive and behavioral function have also been reported.^36^ Treadmill exercise ameliorates impaired glucose metabolism in the posterior cingulate cortex, a metabolically active region of the brain that is involved in a range of cognitive processes, including spatial memory.^25^^,^^37^ It is difficult to determine, however, whether changes in glucose metabolism within the brain lead to improvements in cognition, as exercise targets several pathways that mediate cognitive performance, including neurogenesis and Aβ clearance. The importance of systemic factors released into circulation during exercise is increasingly being appreciated as a means through which exercise may provide metabolic benefits that may be beneficial in diseases such as AD.^38^

Recently, the paradigm of small extracellular vesicle (EV) tissue cross-talk during exercise has gained traction as a pathway through which cargo, including proteins, micro RNA (miRNA), and metabolites, can enter circulation, enabling inter-organ communication independently of classical secretion.^39^ Notably, exercise-released EVs obtained from humans contain proteins involved in energy production, including glycolic enzymes that lack a predicted signal sequence peptide typical of classically secreted proteins.^39^ The functional role of exercise-released EVs in regulating metabolic processes, which may be beneficial in diseases such as AD, has not yet been investigated.^40^ Therefore, the aims of this study were to (1) investigate whether male APP/PS1 mice show signs of metabolic dysfunction and whether any beneficial effects of voluntary exercise training (VET) may be provided, (2) determine whether VET improves cognitive function in the APP/PS1 transgenic mouse (Tg) model of AD,^41^^,^^42^ (3) analysis the proteomic and miRNA profiles within the cargo of exercise and sedentary EVs from mice, and (4) examine whether exercise-released EVs from healthy donor mice administered to APP/PS1Tg recipient mice mediate the benefits in cognition and metabolism observed with VET.

## Results

### Voluntary exercise training normalizes resting energy expenditure in male APP/PS1Tg mice

**Figure 1:**
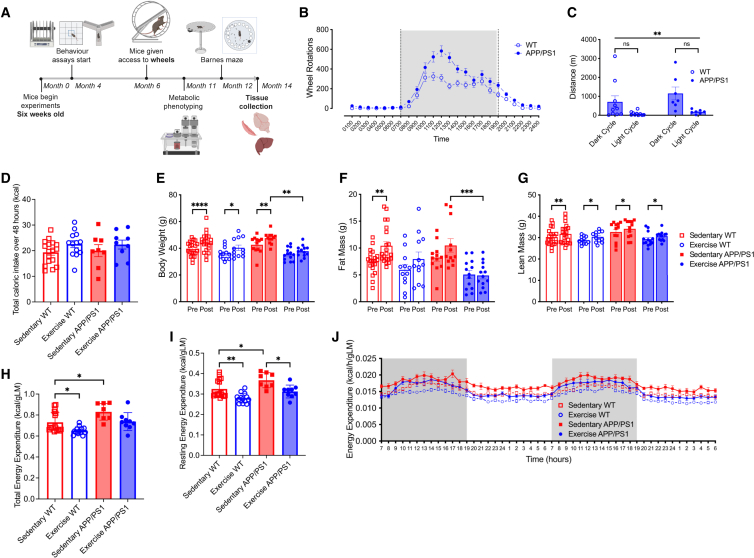
Voluntary exercise training normalizes elevated resting energy expenditure, reduces fat mass, and restores metabolic parameters in male APP/PS1Tg mice to wild-type levels (A) Experimental design. (B) Running wheel data, shown as wheel rotations for a 24-h cycle averaged over three weeks. (C) Total distance over 24 h, averaged over three weeks for both dark and light cycles. (D) Total caloric intake over 48 h during metabolic phenotyping. (E) Body weight. (F) Absolute fat mass. (G) Absolute lean mass; pre-wheels (5 months old) and post-wheels (12 months old). (H and I) (H) Total and (I) resting energy expenditure over same 48 h period, normalized to lean mass. (J) Averaged energy expenditure (hour bins) over same 48-h period, normalized to lean mass. Significance was calculated using two-way/mixed model ANOVA Tukey post hoc, ∗*p* < 0.05, ∗∗*p* < 0.01, ∗∗∗*p* < 0.001, ∗∗∗∗*p* < 0.0001. Sedentary WT *n* = 17–21, exercise WT *n* = 12–13, sedentary APP/PS1 n = 8–12, exercise APP/PS1 n = 9–13. All data are presented as the group mean ± SEM.

Male APP/PS1Tg and/or littermate control (WT) mice were dual housed in a cage with either two locked (sedentary) or two unlocked (exercise) running wheels for 6 months (sedentary WT *n* = 21; exercise WT *n* = 13; sedentary APP/PS1Tg *n* = 12; exercise APP/PS1Tg *n* = 13). To maintain data consistency and avoid potential variations linked to female reproductive cycles, experiments were conducted using male mice. Experimental timeline is shown in Figure 1A. Mice were housed dual in cages because work from our laboratory demonstrated that social isolation affects the physiological response to wheel running.^43^ Wheel rotations over a 24-h cycle averaged over three weeks (wk) showed a similar trend for both APP/PS1Tg and WT mice, with all mice running predominantly during the dark cycle. There were no differences in the distance between APP/PS1Tg and WT mice in either the dark or light cycle (Figures 1B and 1C; RM ANOVA, phase × genotype interaction, *F*_1,30_ = 0.5335, *p* = 0.4708, phase main effect, *F*_1,30_ = 11.14, *p* = 0.0023). VET did not affect food intake when comparing the four groups (Figure 1D; ANOVA, exercise × genotype interaction, *F*_1,43_ = 0.0670, *p* = 0.7970). Body weight increased in all groups except the exercise APP/PS1Tg mice (Figure 1E; RM ANOVA, time × exercise × genotype interaction, *F*_1,54_ = 2.873, *p* = 0.4173; pairwise comparison: pre-post sed WT *p* < 0.0001, pre-post ex WT *p* = 0.0272, pre-post sed APP *p* = 0.0051, pre-post ex APP *p* = 0.5162, post sed APP vs. post ex APP *p* = 0.0011), which was entirely expected as the mice matured during the intervention period. Fat mass only increased the sedentary WT mice; however, VET reduced fat mass in the APP/PS1Tg mice (Figure 1F; RM ANOVA, time × exercise × genotype interaction, *F*_1,54_ = 0.7552, *p* = 0.3887; pairwise comparison: pre-post sed WT *p* = 0.0016, pre-post ex WT *p* = 0.3487, pre-post sed APP *p* = 0.2279, pre-post ex APP *p* > 0.9999, post sed APP vs. post ex APP *p* = 0.0010). Lean mass increased in all groups (Figure 1G; RM ANOVA, time × exercise × genotype interaction, *F*_1,54_ = 0.06259, *p* = 0.8034; pairwise comparison: pre-post sed WT *p* = 0.0011, pre-post ex WT *p* = 0.0139, pre-post sed APP *p* = 0.0234, pre-post ex APP *p* = 0.0277).

At 11 months of age, the mice were metabolically phenotyped using the Promethion metabolic cage system. All mice were acclimatized to single housing for 3 days before being placed in the system and measured for 2 days. APP/PS1Tg sedentary mice had markedly higher (*p* < 0.05) oxygen consumption (VO_2_) and carbon dioxide production (VCO_2_) over the 48 h measurement period compared with the other three groups. Of note, VET in the APP/PS1Tg mice normalized both VO_2_ and VCO_2_ to levels seen in WT mice (Figure S1A). There was no difference in the respiratory quotient (RQ) between the groups (Figure S1A). Since animals performed some VET during the light cycle, it was unsurprising that the time they slept over the 48 h data collection period was decreased in both WT and APP/PS1Tg mice with VET (Figure S1B; ANOVA, exercise × genotype interaction, *F*_1,43_ = 0.4094, *p* = 0.5257; pairwise comparison: sed WT vs. sed APP *p* = 0.0116, sed WT vs. ex WT *p* < 0.0001, sed WT vs. ex APP *p* < 0.0001, sed APP vs. ex WT *p* = 0.0287, sed APP vs. ex APP *p* < 0.0001, ex WT vs. ex APP *p* = 0.0011). TEE was increased in sedentary APP/PS1Tg mice relative to sedentary WT controls. VET decreased TEE in WT mice, while there was a trend for this to occur in APP/PS1Tg mice; however, the results were not significant (Figures 1H and 1J; ANOVA, exercise × genotype interaction, *F*_1,43_ = 0.08152, *p* = 0.7766; pairwise comparison: sed WT vs. sed APP *p* = 0.0224, sed WT vs. ex WT *p* = 0.0397, sed WT vs. ex APP *p* = 0.9961, sed APP vs. ex WT *p* < 0.0001, sed APP vs. ex APP *p* = 0.0814, ex WT vs. ex APP *p* = 0.0642). REE also increased in sedentary APP/PS1Tg mice relative to sedentary WT controls. VET decreased REE independent of genotype (Figure 1I; ANOVA, exercise × genotype interaction, *F*_1,43_ = 0.2417, *p* = 0.6255; pairwise comparison: sed WT vs. sed APP *p* = 0.0344, sed WT vs. ex WT *p* = 0.0073, sed WT vs. ex APP *p* = 0.8332, sed APP vs. ex WT *p* < 0.0001, sed APP vs. ex APP *p* = 0.0129, ex WT vs. ex APP *p* = 0.1678). These data demonstrate that elevated REE, which is associated with cognitive decline in both mice and humans^3^^,^^7^^,^^44^^,^^45^ is normalized in APP/PS1Tg mice after 6 months of VET.

### Voluntary exercise training improves long term memory and reduces amyloid-β accumulation in the cortices but not the hippocampus of APP/PS1Tg mice

**Figure 2:**
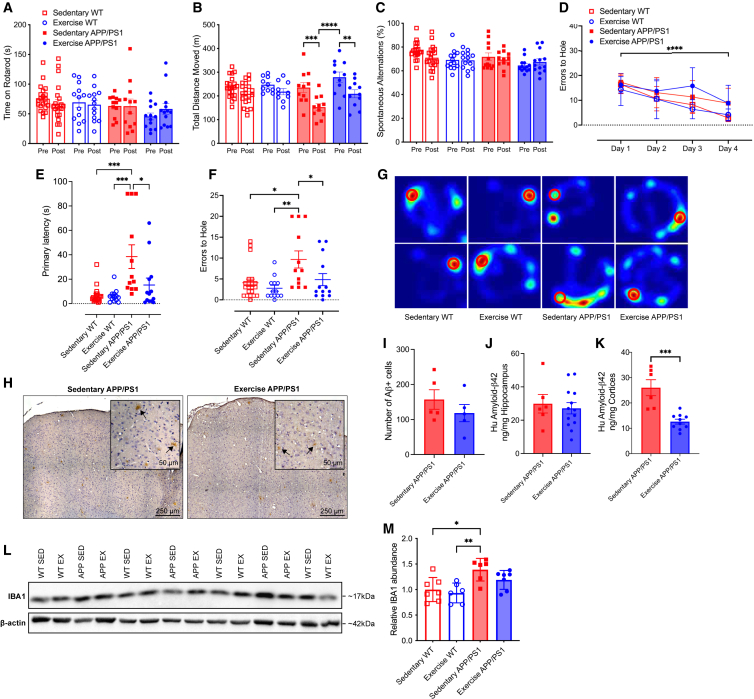
Voluntary exercise training enhances long-term memory in male APP/PS1Tg mice, reduces cortical Aβ accumulation and lowers microglial activation, without affecting short-term memory or motor coordination (A) Rotarod results for all four experimental groups. (B) Total distance moved during locomotor activity test (LMA). (C) Percentage of spontaneous alternations during a spontaneous alternation Y-maze test. (D) Errors to the target hole during the Barnes maze learning phase. (E and F) (E) Primary latency and (F) errors to target hole achieved during probe test conducted using a Barnes maze. (G) Representative heat maps from two mice per group during the probe test of the Barnes maze. The location of the target hole is indicated by red circles. (H) Representative images of DAB-stained cortical regions from sedentary and exercise APP/PS1 mice. DAB staining (brown) indicates the presence of amyloid-beta (Aβ) plaques in the cortex (indicated with black arrows), with hematoxylin counterstain (blue) highlighting cell nuclei. (I) Quantification of Aβ plaque burden across the left cerebral hemisphere in sedentary and exercise APP/PS1 mice. Average number of Aβ-positive cells per section determined by two independent blinded scorers. (J) Protein levels of human amyloid-β42 measured using ELISA in the hippocampus of sedentary and exercised APP/PS1 mice, shown as a ratio to total hippocampal tissue loaded. (K) Protein levels of human amyloid-β42 measured using ELISA in the cortex of sedentary and exercised APP/PS1 mice, shown as a ratio to total cortex tissue loaded. (L) Representative western blot images displaying IBA1 protein expression in brain tissue samples from sedentary and exercise WT and APP/PS1 groups. (M) Quantification of IBA1 protein levels normalized to β-actin and expressed relative to the sedentary WT group. Significance was calculated using two-way ANOVA Tukey post hoc (A–E, M) negative binomial regression Tukey post hoc (F), and unpaired t test (I–K). ∗*p* < 0.05, ∗∗*p* < 0.01, ∗∗∗*p* < 0.001, ∗∗∗∗*p* < 0.0001. Sedentary WT n = 7–21, exercise WT n = 6–13, sedentary APP/PS1 n = 5–12, exercise APP/PS1 n = 4–13. All data are presented as the group mean ± SEM.

To evaluate the effects of VET on motor and cognitive function in the APP/PS1Tg mice, all animals underwent a suite of behavioral tests in the final month of the intervention (Figure 1A). In the initial test, all mice were placed on a rotarod to evaluate balance, grip strength, and motor coordination. There was no effect of genotype or VET on motor coordination as measured by the time spent on the rotarod (Figure 2A; RM ANOVA, time × exercise × genotype interaction, *F*_1,55_ = 0.2340, *p* = 0.6305). Next, we performed a locomotor activity test (LMA) to evaluate changes in ambulatory locomotor activity. Mice were placed into a white box for 90 min, and total distance moved was measured. Total distance moved was not altered during the intervention period in the WT mice, irrespective of VET. In contrast, in sedentary APP/PS1Tg mice, the total distance moved was markedly decreased during the intervention period. While VET tended to increase total distance moved in APP/PS1Tg mice overall, these mice nonetheless moved less after, compared with before, the intervention period (Figure 2B; RM ANOVA, time × exercise × genotype interaction, *F*_1,49_ = 0.2004, *p* = 0.6564; pairwise comparison: pre-post sed APP *p* = 0.0002, pre-post ex APP *p* = 0.0022, pre ex APP vs. post sed APP *p* < 0.0001). Changes in short-term memory were assessed using the spontaneous alternation Y-maze test. Neither VET nor genotype influenced short-term memory and no changes in the percentage of spontaneous alterations were observed during the Y-maze test (Figure 2C; RM ANOVA, time × exercise × genotype interaction, *F*_1,55_ = 0.003216, *p* = 0.9550).

Finally, mice underwent the Barnes maze test, a hippocampal-dependent task in which animals learn the relationship between distal cues in the surrounding environment and a fixed escape location.^46^ We have previously validated this test with a scopolamine intervention test.^22^ All four groups of mice were equally effective in learning the task during the initial 4 days (Figure 2D; RM mixed-effects, time × exercise × genotype interaction, *F*_3,153_ = 1.651, *p* = 0.1801; time main effect, *F*_3,153_ = 54.56, *p* < 0.0001). Time to the target hole was elevated when comparing sedentary APP/PS1Tg with WT mice, indicating a long-term memory decline in the APP/PS1Tg mice. Importantly, VET decreased the time taken to the correct hole in the APP/PS1Tg mice, demonstrating that VET improved cognitive decline in our mouse model of AD (Figure 2E; ANOVA, exercise × genotype interaction, *F*_3,54_ = 8.740, *p* < 0.0001; pairwise comparison: sed WT vs. sed APP *p* < 0.0001, sed APP vs. ex WT *p* = 0.0004, sed APP vs. ex APP *p* = 0.0146). These results were supported by the errors to hole with the sedentary APP/PS1Tg mice also showing an increase in the number of errors made before finding the target hole compared with the WT and exercise APP/PS1Tg group (Figure 2F; Negative binomial, exercise × genotype interaction, *F*_1,55_ = 0.4126, *p* = 0.5207; pairwise comparison: sed WT vs. ex WT *p* = 0.6276, sed WT vs. sed APP *p* = 0.0227, ex WT vs. sed APP *p* = 0.0020, sed APP vs. ex APP *p* = 0.0461). Representative location heat maps (longer time spent indicated in red) during the probe test for two mice per group, with the target hole indicated by a red circle, are shown in Figure 2G. As an added measure, we also evaluated the distance traveled during the Barnes maze test. The distance traveled during the test was less in the sedentary APP/PS1Tg mice compared with the other three groups (Figure S1C; ANOVA, exercise × genotype interaction, *F*_1,54_ = 0.2101, *p* = 0.2101; pairwise comparison: sed WT vs. sed APP *p* = 0.0010, sed APP vs. ex WT *p* < 0.0001, sed APP vs. ex APP *p* = 0.0014), which is the inverse of the pattern observed with the error-to-hole measure. This result may be attributed to the sedentary APP/PS1Tg mice displaying impaired spatial memory, hindering their ability to remember the target hole’s location. Instead, they tended to remain stationary at any hole for the duration of the probe test. In contrast, WT and exercise APP/PS1Tg mice tended to find the target hole location quickly and upon realizing there was no longer a safe tunnel, begin exploring the rest of the hole locations, leading to an increase in distance traveled. Path length (distance from starting box to target hole) was not significantly different between groups; however, three sedentary APP/PS1Tg mice did not find the target hole during the probe trial and therefore were excluded (Figure S1D; ANOVA, exercise × genotype interaction, *F*_1,50_ = 0.2717, *p* = 0.6045). Primary latency normalized to path length to control for potential differences in ambulation showed a similar pattern to the results observed for primary latency and errors to the hole, with the sedentary APP/PS1Tg group significantly higher compared with both WT groups and the exercise APP/PS1Tg group. Although no significant interaction, there were main effects for both exercise and genotype (Figure S1E; ANOVA, exercise × genotype interaction, *F*_1,49_ = 2.316, *p* = 0.1345, exercise main effect: *F*_1,49_ = 6.688, *p* = 0.0127, genotype main effect: *F*_1,49_ = 12.18, *p* = 0.0010; pairwise comparison: sed WT vs. sed APP *p* = 0.0015, sed APP vs. ex WT *p* = 0.0004, sed APP vs. ex APP *p* = 0.0129).

To explore whether the enhancements in long-term memory were linked to alterations in amyloid-beta levels (Aβ), we assessed the total number of amyloid-beta positive plagues within the cortex and hippocampus and concentration of human Aβ42 in the hippocampus and cortices of both the exercise and sedentary APP/PS1Tg mice at study endpoint. As anticipated, human Aβ42 was not detectable in the WT mice. Although there was no significant difference in the number of Aβ positive cells, the number tended lower in the exercise APP/PS1Tg group (representative images shown in Figure 2H. Quantified in Figure 2I; unpaired t test t(8) = 1.043, *p =* 0.3274). There was no significant difference in the Aβ42 concentration in the hippocampus between the sedentary and VET groups (Figure 2J; unpaired t test, t(17) = 0.4383, *p* = 0.6667). However, a marked difference was observed in the cortices of sedentary and VET APP/PS1Tg mice (Figure 2K; unpaired t test, t(14) = 4.968, *p* = 0.0002). This divergence may stem from the characteristics of the APP/PS1Tg mouse model. Although it is extensively used to study amyloid pathology linked to AD, it predominantly displays features of cerebral amyloid angiopathy.^47^ This is characterized by the deposition of Aβ in the cerebral arterial walls, leading to vascular fragility and cognitive decline. As a result, the APP/PS1Tg mouse model might be especially sensitive to the benefits of VET in the cortices rather than the hippocampus, due to the positive impact of exercise training on vascular health in the brain, angiogenesis, and glymphatic clearance.^48^^,^^49^^,^^50^ Additionally, exercise is known to reduce neuroinflammation in addition to the range of benefits it elicits on peripheral health.^51^ To assess whether exercise reduced neuroinflammation we assessed levels of IBA1, a marker of microglia activation, tumor necrosis factor alpha (TNFα) and interleukin (IL)-1β via western blot. Sedentary APP/PS1Tg mice had significantly increased levels of IBA1 compared with the WT groups; however, although levels in the exercise APP/PS1Tg mice tended lower, this was not significant (representative blots shown in Figure 2L; quantification Figure 2M; ANOVA, exercise × genotype interaction, *F*_1,22_ = 0.4313, *p* = 0.4313; pairwise comparison: sed WT vs. sed APP *p* = 0.0153, sed APP vs. ex WT *p* = 0.0057, sed APP vs. ex APP *p* = 0.3452). Other markers of inflammation assessed including TNFα (Figures S1F and S1G; ANOVA, exercise × genotype interaction, *F*_1,22_ = 0.6960, *p* = 0.4131) and the precursor and mature form of IL-1β (Figures S1H and S1I; pro-IL-1β: ANOVA, exercise × genotype interaction, *F*_1,22_ = 0.2446, *p* = 0.6258. IL-1β p17: ANOVA, exercise × genotype interaction, *F*_1,22_ = 0.3181, *p* = 0.5785), did not show significant differences in protein abundance between groups. Finally, we assessed several key markers of metabolic homeostasis, blood glucose, insulin and NEFAs. No differences in blood glucose or NEFAs were observed between any groups (Figure S2A; blood-glucose: ANOVA, *F*_1,15_ = 0.01193, *p* = 0.9145; Figure S2C; NEFAs: ANOVA, *F*_1,31_ = 0.0919, *p* = 0.7638). Blood insulin levels were significantly decreased in the exercise APP/PS1Tg group compared with sedentary APP/PS1Tg group and there was a significant exercise main effect (Figure S2B; ANOVA, *F*_1,30_ = 2.880, *p* = 0.1000, exercise main effect: *F*_1,30_ = 8.055, *p* = 0.0081; pairwise comparison: sed APP vs. ex APP *p* = 0.0378). Taken together, these data indicate that VET improves long-term memory in our mouse model of AD, potentially by reducing accumulation of Aβ42 in the cortex and reducing microglial activation, a marker of neuroinflammation. As expected, VET also improved systemic metabolic markers, with an increase in insulin sensitivity observed.

### Acute treadmill exercise in C57BL/6 mice releases EVs, which express tetraspanin proteins consistent with small EVs

**Figure 3:**
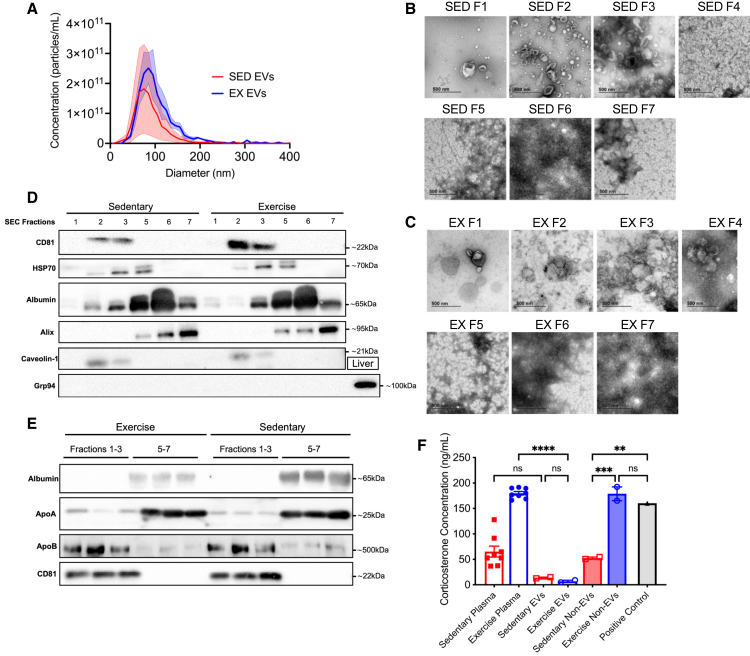
Acute treadmill exercise in C57BL/6 mice releases extracellular vesicles expressing tetraspanin proteins consistent with markers of small EVs (A) Size distribution acquired by ZetaView of exercise and sedentary EVs isolated from single mice using SEC (*n* = 3 per condition). (B) Transmission electron microscopy (TEM) for each fraction isolated from sedentary mice plasma using the qEV10/35 nm column. (C) TEM for each fraction isolated from exercise mice plasma using SEC. (D) Western blot for various antibodies on individual SEC fractions from exercise and sedentary mouse plasma. (E) Western blot for various antibodies on pooled fractions from EVs isolated from exercise and sedentary mouse plasma. EV-rich (1–3) and non-EV (5–7) SEC fractions were pooled and concentrated using Amicon Ultra-15 Centrifugal Filter Units. Three replicates for each of the pooled fractions. (F) Concentration of corticosterone in sedentary and exercise plasma, EVs, and non-EV fractions isolated via SEC, and positive control consisting of plasma from mice restrained for 2 h. ∗∗*p* < 0.01, ∗∗∗*p* < 0.001, ∗∗∗∗*p* < 0.0001. Sedentary plasma n = 8, exercise plasma n = 8, sedentary EVs n = 2, exercise EVs n = 2, sedentary non-EVs n = 2, exercise non-EVs n = 2. All data are presented as the group mean ± SEM.

We next examined the possible mechanisms underlying the protective effect of VET on energy expenditure and long-term memory. Several proteins and miRNAs have been observed in exercise-induced EVs that may be implicated in protection against dementia (for review, see study by Fuller et al.^40^). Before investigating whether exercise-released EVs are involved in the cognitive and metabolic benefits observed in mice who undertake voluntary running, we first deeply profiled the mouse EVs as our previous work with EV’s was conducted in humans.^39^ EVs were isolated from the plasma of C57BL/6 mice after 60 min of treadmill exercise (eEVs) and sedentary control (sEVs) mice by size exclusion chromatography (SEC). EVs were isolated via SEC using IZON qEV10 35 nm columns (IZON Science, NZ) and the EV-rich fractions (fractions 1–3) were pooled and concentrated (AMICON Ultra filters). Particle size and concentration distributions were similar between the sEVs and eEVs (ZetaView, Particle Metrix, Germany) (Figure 3A). This result highlights a notable distinction in the size distribution and particle concentration of EVs isolated using SEC, compared with other methods such as UC, the latter being employed in our previous work.^39^ In this study, we show a 2-fold increase in EV concentration following acute exercise. This observation was further corroborated by our recent investigations using the ExoView R100 Platform (Unchained Labs, CA, USA),^52^ an unbiased technique for measuring EV concentration in plasma, which also indicated a similar 1.5- to 2-fold increase in circulating EVs post-exercise. However, significant differences in concentration were not observed when using SEC isolation. This can be attributed to the normalizing effect of the SEC process, where the pores of the spherical beads in the SEC gel, become saturated with EVs. The excess EVs, not captured by these pores, pass through. Consequently, when starting with a material such as plasma that inherently contains a high number of EVs, even under sedentary conditions, the resulting concentration tends to be similar between conditions. Transmission electron microscopy (TEM) of each fraction showed considerable non-particular contamination in fractions 4–7 in both the sEV and eEV SEC isolates (Figures 3B and 3C). Therefore, for subsequent functional studies, we pooled fractions 1–3 in order to minimize plasma protein contamination. To confirm the presence of EVs in the absence of contaminating plasma proteins and lipoproteins (a common co-elute when using SEC for EV isolation), western blot analysis was conducted with known EV and small EV markers (CD81, HSP70, and caveolin-1) along with albumin, glucose-regulated protein 94 (Grp94), ApoA1, and ApoB were performed on the individual SEC fractions (Figure 3D), and pooled EV-rich (fractions 1–3) and EV-poor (fractions 5–7) (Figure 3E). The tetraspanin protein CD81, commonly enriched in the membranes of small EVs (∼50–100 nm in diameter), was present in fractions 2 and 3 in both sEVs and eEVs isolates (Figure 3D). Heat shock protein (HSP) 70, another marker enriched in larger EVs (∼1,000 nm in diameter), was present in fractions 2–5. Albumin was present in fractions 2–7 in both sEVs and eEVs but was highly enriched in fractions 5 and 6. ALIX, a protein involved in EV biogenesis and a number of other intracellular functions, such as apoptosis, has been used as a marker of EVs; however, in our isolates, it was not present in the EV-rich fractions, but rather in the EV-poor fractions. This may be due to cellular contamination, eluting later in the column, as ALIX is a cytoplasmic protein ubiquitously expressed in most cells. Caveolin-1, a structural protein of caveolae that is involved in the biogenesis and cargo selection of larger EVs, such as microvesicles (MVs), was present in the EV-rich fractions of both sEVs and eEVs. Grp94 is involved in the folding and assembly of proteins secreted from cells and is typically absent in EVs. A sample of liver tissue from a non-treated WT mouse was included as a positive control, with no Grp94 present in the EV-rich or EV-poor fractions. Western blot (WB) analysis of the pooled EV-rich and EV-poor fractions is shown in Figure 3E. Albumin was not present in the EV-rich fractions of either sEV or eEV isolates. Lipoprotein contamination is a common limitation of SEC-isolated EVs. Although ApoA was highly expressed in the EV-poor fractions, ApoB was present in the EV-rich fractions. Importantly, there appeared to be no difference in the amount of ApoB present between the sEV and eEV, based on the same total protein being loaded and using CD81 as a proxy for equal concentration of EVs loaded. As expected, CD81 was present only in the pooled EV-rich fractions (Figure 3E). To investigate the potential effects of factors such as hormones, which may co-elute owing to their similar size or via lipoprotein rafts, the concentrations of the anti-inflammatory cytokine IL-10, pro-inflammatory cytokine IL-1β, and corticosterone/cortisol were assessed using ELISA to ensure that the EV isolation method removed contaminants that may alter metabolic function. IL-10 levels were not detectable in any of the fractions and did not differ in either sedentary or exercise plasma (data not shown). IL-1β was not detectable in EV-rich or EV-poor fractions and displayed large biological variation in the plasma of sedentary and exercise mice, with no difference between them (data not shown). Corticosterone/cortisol levels were higher (*p* < 0.01) in exercise plasma than in sedentary plasma and were similar to those found in the exercise EV-poor fraction (Figure 3F; ANOVA, treatment, *F*_6,18_ = 44.40, *p* < 0.0001). Corticosterone/cortisol levels in the exercise plasma and EV-poor fractions were comparable to those observed in the mouse plasma after 2 h of restraint (positive control). Importantly, the concentration of corticosterone/cortisol was low in the EV-rich fractions and did not differ between sedentary and exercise isolates. Together, these results demonstrate EVs isolated from mouse plasma using SEC, display markers consistent with small EVs and effectively removes potential confounding factors such as corticosterone/cortisol ensuring the robustness of the findings presented in this work.

### sEVs and eEVs obtained from C57BL/6 mice differ in their miRNA and proteomic profile

**Figure 4:**
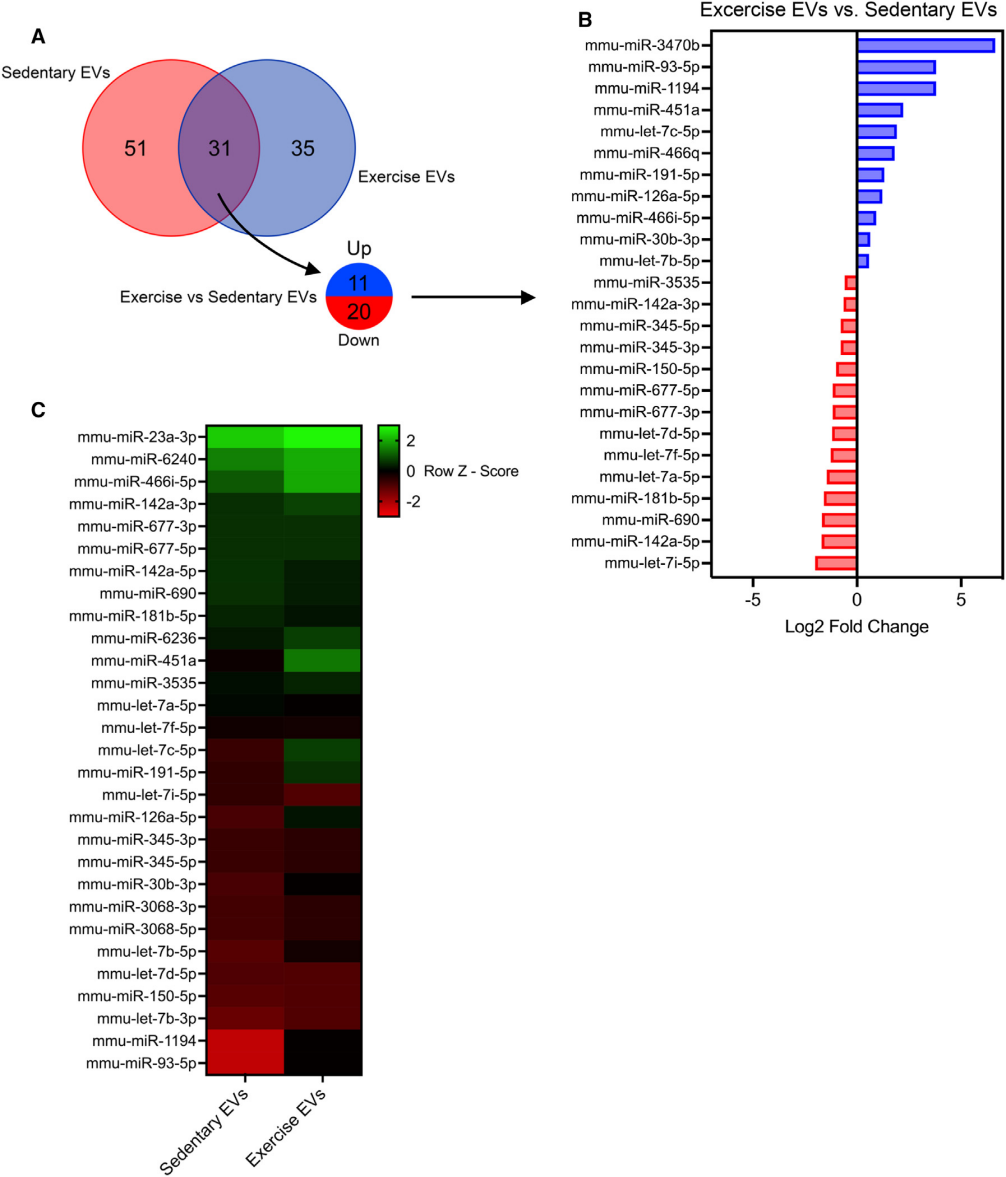
Exercise-released extracellular vesicles display a distinct miRNA profile compared to sedentary controls, revealing unique miRNA species that may influence processes relevant to cognitive function and Alzheimer’s disease pathology (A) Venn diagram showing the overlap between differentially expressed EV miRNAs between sedentary and exercise. (B) Top 25 differentially regulated miRNAs (>log2 FC 0.5), upregulated (blue), and downregulated (red) in exercise EVs. (C) Heatmap depicting the differential expression of EV miRNAs in exercise EVs vs. sedentary EVs. Rows correspond to individual miRNAs and their relative expression levels. Red indicates expression levels lower than the mean, whereas green indicates expression levels higher than the mean.

To investigate whether exercise and sedentary EVs differed in their cargo and whether potential differences may be responsible for distinctive biological functions, we performed miRNA sequencing and proteomic analysis of EVs from sEVs and eEVs. For miRNA sequencing, RNA was extracted from EVs isolated using SEC from plasma pooled from approximately 20 mice. In total, 117 miRNA species were detected in circulating EVs (51 unique to sedentary EVs, 35 unique to exercise EVs, and 31 that appeared in both; Figure 4A). Of the 31 miRNA species present in both sEVs and eEVs, 11 were upregulated in eEVs and 20 were downregulated in sEVs. The top 25 differentially expressed species (>log2 fold change (FC) 0.5) are shown in Figure 4B, with species upregulated shown in blue and downregulated species in red in eEVs. The top 30 most differentially expressed species relative to the average expression level of all miRNA species (*Z* score) are shown in Figure 4C. Rows correspond to individual miRNAs and their relative expression levels. Red indicates expression levels lower than the mean, whereas green indicates expression levels higher than the mean. We identified several miRNA candidates differentially expressed when comparing sEVs with eEVs (as measured by both FC and *Z* score), which have been implicated in a range of biological processes that may be relevant in diseases such as AD.

**Figure 5:**
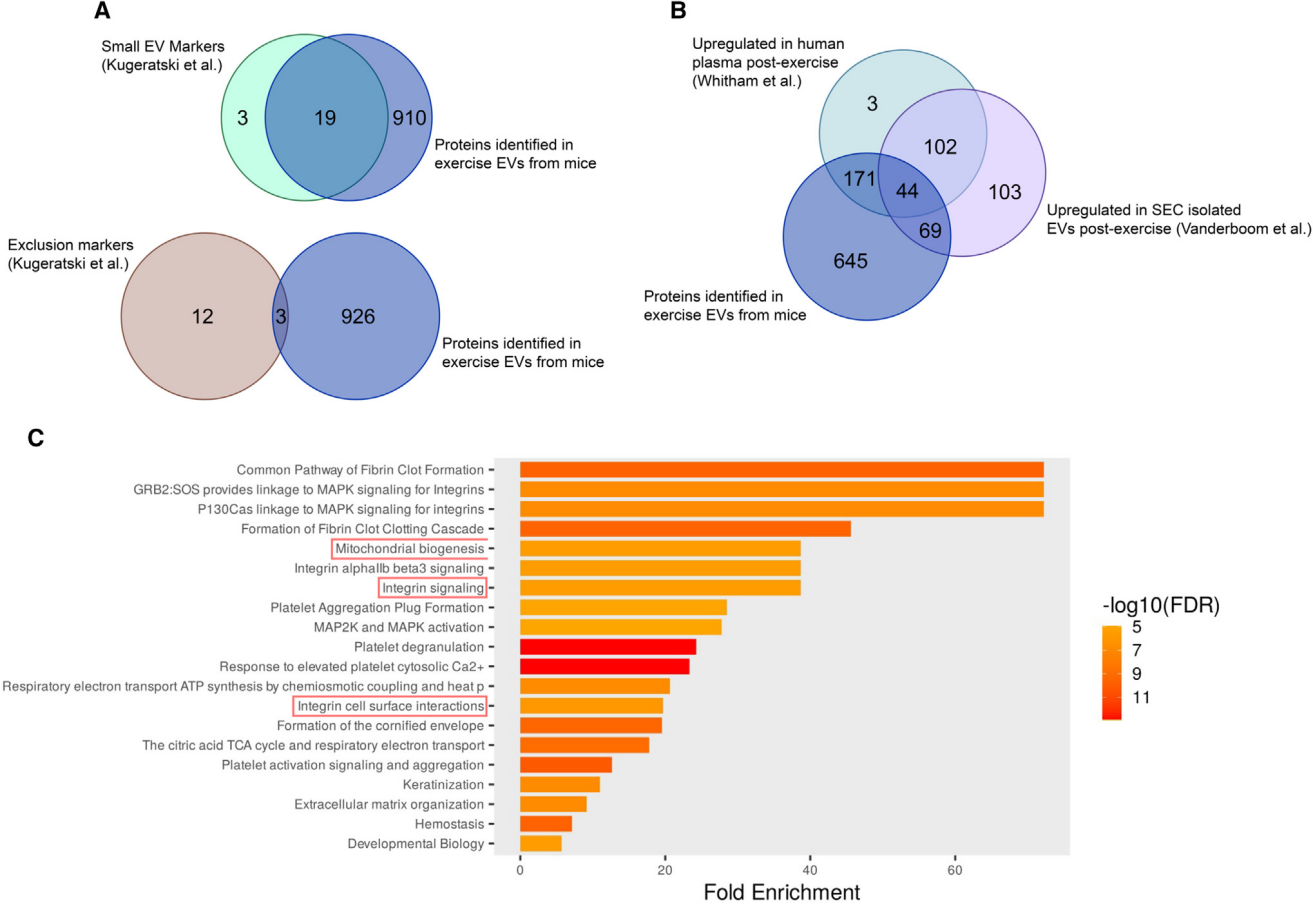
Exercise-released extracellular vesicles are enriched in proteins linked to mitochondrial biogenesis and integrin signaling, highlighting potential pathways relevant to Alzheimer’s disease and neuronal EV uptake (A) Top: Venn diagram showing the overlap between proteins identified in mouse EVs from exercise plasma and markers associated with small EVs, and bottom: Venn diagram showing overlap between proteins identified in EVs and exclusion markers.^53^ (B) Venn diagram showing the overlap between proteins identified in mouse EVs from exercise plasma, proteins upregulated in human plasma post-exercise^39^ and in EVs isolated from human plasma post-exercise.^54^ (C) Pathway enrichment analysis performed on the 84 significantly differentially expressed proteins identified in exercise EVs compared with sedentary EVs. Pathways enriched in exercise EVs that may regulate processes involved in metabolism and drive tropism are highlighted in red. Sedentary EVs were considered controls, and exercise EVs were the condition of interest. The size and color of the horizontal bars indicate the scale of enrichment and level of significance (logarithm of the adjusted *p* value [FDR], orange to red).

To confirm the presence of EVs and explore potential protein cargo that may drive processes relevant to diseases such as AD, we performed proteomic analysis of sEVs and eEVs. Overall, we identified 929 proteins in eEVs (84 that were significantly increased compared with sEVs), which were highly enriched in markers associated with EVs (19/22) and low in levels of exclusion markers (3/15),^53^ indicating adequate isolation of EVs (Figure 5A). While there was poor agreement between the 84 significantly differing proteins within the eEVs and human plasma and EVs datasets (data not shown), there was sufficient agreement when comparing all proteins identified in mouse eEVs and those upregulated in human plasma post-exercise 39 and human EVs isolated from plasma post-exercise^54^ (Figure 5B). Pathway enrichment analysis of proteins significantly different between eEVs and sEVs, employing the total mouse genome as a background, (Figure 5C) indicated several pathways that may be relevant in AD, including mitochondrial biogenesis as dysfunctional mitochondria are believed to play a crucial role in the development of AD^55^ and integrin signaling/cell surface interactions, which have been shown to drive EV tropism.^56^ In addition, integrin-mediated interactions have been implicated in facilitating neuronal uptake of EVs; however, further work is needed to determine if exercise-induced EVs exhibit these integrins and if they are required for neuronal uptake.

### Intranasal delivery of eEVs, but not sEVs, from C57BL/6 mice into APP/PS1Tg mice decreases whole body energy expenditure but does not improve long-term memory or change amyloid-β accumulation in the hippocampus or cortex of APP/PS1Tg mice

**Figure 6:**
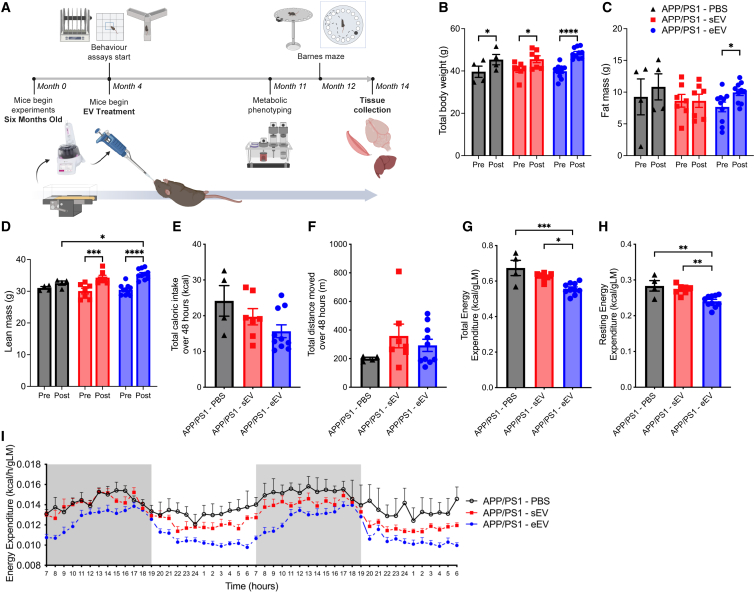
Intranasal delivery of exercise-released extracellular vesicles reduces resting and total energy expenditure in APP/PS1Tg mice, recapitulating the metabolic effects of voluntary exercise training (A) Experimental design. (B) Body weight. (C) Absolute fat mass. (D) Absolute lean mass; pre-treatment (4 months old) and post-treatment (12 months old). (E) Total caloric intake over 48 h during metabolic phenotyping. (F) Total distance moved over 48 h during metabolic phenotyping. (G and H) (G) Total and (H) resting energy expenditure over the same 48 h period, normalized to lean mass. (I) Averaged energy expenditure (hour bins) over same 48-h period, normalized to lean mass. Significance was calculated using two-way/mixed model ANOVA Tukey post hoc, ∗*p* < 0.05, ∗∗*p* < 0.01, ∗∗∗*p* < 0.001, ∗∗∗∗*p* < 0.0001. APP/PS1 PBS, *n* = 4; APP/PS1 sEV, *n* = 7; APP/PS1 eEV, *n* = 10. All data are presented as the group mean ± SEM.

It has previously been reported that delivery of EVs to the brain via the nasal route can result in delivery of EVs to several regions of the brain, including the hippocampus.^57^ Accordingly, to determine whether EVs are a mechanism by which VET mediates the beneficial effects on energy expenditure and long-term memory, we performed intranasal delivery of 20 μg of sEV and eEV from C57BL/6 donor mice (suspended in PBS), along with PBS as a control, into APP/PS1Tg mice once per week for 8 months (experimental timeline Figure 6A). As in the initial experiment, body weight increased in all groups (Figure 6B; RM ANOVA, time × treatment interaction, *F*_2,18_ = 1.329, *p* = 0.2896; pairwise comparison: pre-post PBS *p* = 0.0292, pre-post sEV *p* = 0.0162, pre-post eEV *p* < 0.0001). Lean mass increased in the sEV and eEV groups but not the PBS treated group (Figure 6D; RM ANOVA, time × treatment interaction, *F*_2,18_ = 3.248, *p* = 0.0625; pairwise comparison: pre-post PBS *p* = 0.1965, pre-post sEV *p* = 0.0001, pre-post eEV *p* < 0.0001, post PBS post eEV *p* = 0.0104). Interestingly, however, only the group that received eEVs showed an increase in fat mass over the intervention period, potentially due to the reduction in REE (Figure 6C; RM ANOVA, time × treatment interaction, *F*_2,18_ = 1.507, *p* = 0.2483; pairwise comparison: pre-post eEV *p* = 0.0151). As per the exercise intervention experiment, at 11 months of age, the mice were metabolically phenotyped using the Promethion metabolic cage system. VO_2_, VCO_2_, and RQ measured over the 48 h period did not differ among the three groups (Figure S3A). Total caloric intake (Figure 6E; ANOVA, *F*_2,18_ = 2.705, *p* = 0.0940), total distance moved over 24 h (Figure 6F; ANOVA, *F*_2,18_ = 1.267, *p* = 0.3056), and time spent sleeping (Figure S3B; ANOVA, *F*_2,18_ = 2.248, *p* = 0.1344) were not significantly different among the three groups. Importantly, however, while TEE and REE were not different when comparing PBS control with sEV, delivery of eEV into APP/PS1Tg mice reduced both TEE (Figure 6G; ANOVA, *F*_2,18_ = 11.78, *p* = 0.0005; pairwise comparison: PBS vs. sEV *p* = 0.2074, PBS vs. eEV *p* = 0.0007, sEV vs. eEV *p* = 0.0134) and REE (Figure 6H; ANOVA, *F*_2,18_ = 10.781, *p* = 0.0008; pairwise comparison: PBS vs. sEV *p* = 0.6591, PBS vs. eEV *p* = 0.0025, sEV vs. eEV *p* = 0.0050) (energy expenditure over 48 h shown in Figure 6I) in a manner analogous to that observed with VET in APP/PS1Tg mice.

**Figure 7:**
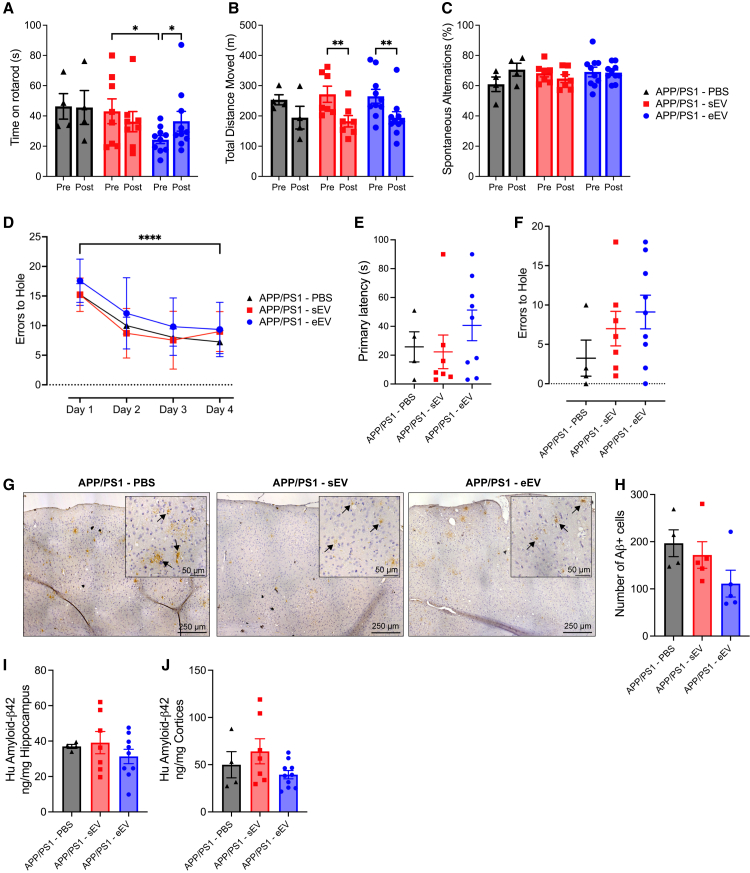
Intranasal delivery of exercise-released extracellular vesicles improves metabolic parameters but does not enhance cognition or reduce Aβ accumulation in APP/PS1Tg mice (A) Total time spent on rotarod. (B) Total distance moved during locomotor activity test (LMA), pre-treatment (4 months old), and post-treatment (12 months old). (C) Percentage of spontaneous alternations during a spontaneous alternation Y-maze test. (D) Errors to the target hole during the Barnes maze learning phase. (E and F) (E) Primary latency and (F) errors to target hole achieved during probe test conducted during the Barnes maze text. (G) Representative images of DAB-stained cortical regions from PBS and EV treated APP/PS1 mice. DAB staining (brown) indicates the presence of Aβ plaques in the cortex (indicated with black arrows), with hematoxylin counterstain (blue) highlighting cell nuclei. (H) Quantification of Aβ plaque burden across the left cerebral hemisphere in PBS and EV treated APP/PS1 mice. (I) Protein levels of human amyloid-β42 measured using ELISA in the hippocampus of APP/PS1 mice treated with PBS and EVs, shown as a ratio to total hippocampal tissue loaded. (J) Protein levels of human amyloid-β42 measured using ELISA in the hippocampus of APP/PS1 mice treated with PBS and EVs, shown as a ratio to total cortex tissue loaded. Significance was calculated using repeated measures two-way ANOVA Tukey post hoc (A–E), negative binomial regression Tukey post hoc (F) and one-way ANOVA Tukey post hoc (H–J) ∗*p* < 0.05, ∗∗*p* < 0.01, ∗∗∗∗*p* < 0.0001. APP/PS1 PBS, *n* = 4; APP/PS1 sEV, *n* = 7; APP/PS1 eEV, *n* = 10. All data are presented as the group mean ± SEM.

As with the initial exercise intervention study, all animals in the EV transfer experiment underwent the suite of behavioral tests pre and post intervention (Figure 6A). Differences were only observed in the eEV group for time on the rotarod (Figure 7A; RM ANOVA, time × treatment interaction, *F*_2,19_ = 3.771, *p* = 0.0418; pairwise comparison: pre-post eEV *p* = 0.0184, pre sEV vs. pre eEV *p* = 0.0396). All groups showed a decrease in the total distance moved in the LMA test, but the results were only statistically significant for the sEV and eEV groups (Figure 7B; RM ANOVA, time × treatment interaction, *F*_2,18_ = 0.2267, *p* = 0.7994; pairwise comparison: pre-post sEV *p* = 0.0058, pre-post eEV *p* = 0.0081). No differences were observed for any group during the spontaneous alternations in the Y-maze test (Figure 7C; RM ANOVA, time × treatment interaction, *F*_2,18_ = 2.304, *p* = 0.1285). All groups learned the Barnes maze task with equal efficiency (Figure 7D; RM ANOVA, time × treatment interaction, *F*_6,54_ = 0.3102, *p* = 0.9290; time main effect, *F*_2.472,44.50_ = 19.04, *p* < 0.0001), and no differences were observed in either time to hole (Figure 7E; ANOVA, *F*_2,17_ = 0.8271, *p* = 0.4542) or error to hole in the Barnes maze test (Figure 7F; negative binomial, *F*_2,17_ = 1.5876, *p* = 0.2044). As expected, no differences were observed in path length (Figure S3C; ANOVA, *F*_2,18_ = 0.9171, *p* = 0.4176) or primary latency normalized to path length either (Figure S3D; ANOVA, *F*_2,18_ = 0.1321, *p* = 0.8771).

We also assessed the total number of Aβ plagues within a single half hemisphere brain section and the concentration of human Aβ42 in the hippocampus and cortices of APP/PS1Tg mice treated with PBS and EVs. There was no significant difference in the number of Aβ plagues between groups, though the mice treated with eEVs tended lower (Figure 7G and 7H; ANOVA, *F*_2,11_ = 2.361, *p* = 0.1403; pairwise comparison: PBS vs. sEV *p* = 0.8225, PBS vs. eEV *p* = 0.1418, sEV vs. eEV *p* = 0.3033). Aβ42 concentration in the hippocampus did not differ between the three groups (Figure 7I; ANOVA, *F*_2,17_ = 0.7539, *p* = 0.4856), or in the cortices either, however a potential trend was observed in the mice treated with eEVs, showing a minor decrease compared with the sEV group (Figure 7J; ANOVA, *F*_2,17_ = 1.994, *p* = 0.1651; pairwise comparison: PBS vs. sEV *p* = 0.6471, PBS vs. eEV *p* = 0.7610, sEV vs. eEV *p* = 0.1417). Finally, we assessed the same key indicators of metabolic homeostasis in the EV treated mice as the VET groups. Interestingly, APP/PS1Tg treated with eEVs showed an increase in blood-glucose levels compared with APP/PS1Tg sEVs (Figure S3E; unpaired t test, t(8) = 2.584, *p* = 0.0324), with no significant differences in insulin or NEFAs (insulin: Figure S3F; unpaired t test, t(9) = 0.6159, *p* = 0.5532. NEFAs: Figure S3G; unpaired t test, t(13) = 0.1010, *p* = 0.9211). Taken together, these data suggest that while the transfer of eEV improved energy metabolism in the APP/PS1Tg mice, this did not translate into any improvements in cognition or significant differences in accumulation of Aβ in the brains of APP/PS1Tg mice treated with eEVs.

## Discussion

Exercise training has been shown to improve cognition and reduce amyloid-beta accumulation in mouse models of AD.^58^^,^^59^ Here, we confirm these previous data, since VET completely normalized the increase in errors to holes in the APP/PS1Tg mice in the Barnes maze test and reduced the accumulation of Aβ42 in the cortices. Moreover, we demonstrate that elevated REE, a suggested mechanism for cognitive decline in both humans with AD and in AD mouse models, is also normalized by VET in the APP/PS1Tg mice. We demonstrate, for the first time, the transfer of metabolic benefits via exercise-released EVs in an APP/PS1Tg mouse model of AD, since the transfer of eEV, but not sEV, decreased TEE and REE. However, it must be noted that this improvement in energy expenditure did not cf. a benefit on cognition.

While the mechanisms underlying the increase in energy requirements in individuals with AD have not been determined, previous studies have suggested that a number of factors, including hypothalamic dysfunction,^60^ malabsorption due to alterations in the gut microbiome, and compensatory hypermetabolism, may be responsible for the increase in peripheral REE. Hypermetabolism in certain brain regions, particularly early in disease progression, may occur in response to neuronal damage. Increases in energy production have been noted in cortical regions, which are minimally affected in the early stages of AD.^61^ This may counteract hypometabolism in the temporoparietal cortical association areas, which occurs long before Aβ pathology or archetypal memory loss arises.^62^ Significantly, we show VET restores REE to WT levels, independent of changes in energy intake. Although it is difficult to assess the role changes in brain metabolism may have on cognitive function since exercise targets a number of pathways that provide benefits, including increased neurogenesis and enhanced clearance of Aβ pathology,^21^ regulation of energy production through exercise leading to reductions in REE may indicate reduced hypometabolism and thus less neuronal damage.

To investigate the potential mechanisms underlying the metabolic benefits demonstrated in APP/PS1 mice during voluntary running, we explored whether EVs could provide a means through which physical activity can mediate biological processes in recipient tissues. Ever since the identification of skeletal muscle as an endocrine organ capable of secreting cytokines, termed “myokines” that can regulate biological functions in recipient tissues, the number of identified myokines has grown substantially,^38^^,^^63^^,^^64^^,^^65^ with several found to be increased within the brain after exercise. These include fibronectin type III domain-containing protein-5 (FNDC5)/irisin, cathepsin B (CTSB), and L-lactate.^66^^,^^67^^,^^68^ Although irisin and CTSB have been implicated in adult hippocampal neurogenesis in mice, further studies are required to establish their role in improving cognition and memory, and whether they would be beneficial for neurodegenerative diseases such as AD in humans. Recently, the concept of EV-mediated tissue cross-talk during exercise was introduced in two landmark studies that demonstrated in healthy human subjects, the rapid release of EVs during an acute high-intensity bout of cycling, which can be taken up by recipient tissues including the liver and spleen.^39^^,^^69^ Several studies have indicated that circulating EVs released during exercise have protective effects on the heart and can regulate endothelial cell function.^70^^,^^71^ However, whether exercise-released EVs mediate biological processes that would benefit diseases such as AD has not yet been investigated. To our knowledge, this is the first study to demonstrate the ability of exercise-released EVs to ameliorate AD-induced metabolic dysfunction. Although the precise mechanisms underlying these benefits warrant further investigation, through miRNA sequencing and proteomic analysis, we identified distinct miRNA and protein profiles in EVs derived from sedentary and exercise donor mice. Specifically, 117 miRNA species were detected, with unique and shared species across sedentary and exercise EVs, highlighting the role of physical activity in modulating EV cargo. mmu-miR-451 was upregulated (4.64 FC) in eEVs, compared with sEVs, and changes in mmu-miR-451 expression have been implicated in glioblastoma, with mmu-miR-451 shown to regulate glioma cell behavior based on glucose levels.^72^ Notably, high-glucose environments have been shown to increase mmu-miR-451 expression and promote growth, while low-glucose levels decrease mmu-miR-451 levels, causing a slowdown in cell growth but an increase in cell migration and survival. This helps cells endure metabolic stress and find better growth conditions by controlling the LKB/AMPK pathway.^72^ Although this is detrimental in glioblastoma, with higher mmu-miR-451 expression linked to shorter survival, it may be beneficial in AD, as in the early stages of AD, there is region-specific hypometabolism; therefore, delivery of mmu-miR-451 via EVs may help regulate neuronal energy availability and could promote cell survival in vulnerable cell populations. Another candidate, mmu-miR-122, was found only in sEVs. Overexpression of mmu-miR-122 has been implicated in delaying neuronal maturation in the brains of developing mice and appears to be upregulated in radiation-induced brain injury.^73^ Intranasal administration of antagomir-122 before irradiation alleviated radiation-induced cognitive impairment, neuronal injury, and neuroinflammation and reduced the levels of pro-inflammatory cytokines released by microglia.^58^ Although delivery of sEVs may not be physiologically applicable, it suggests that the detrimental effects of a sedentary lifestyle may be transferred via circulating EVs and could have harmful effects on the brain by promoting neuroinflammation. Furthermore, mmu-miR-122 also appears to regulate cell metabolism, with cancer cell-derived mmu-miR-122 suppressing glucose uptake in niche cells by downregulating pyruvate kinase and inhibition of mmu-miR-122, restoring glucose uptake in tissues.^74^ mmu-miR-191 was found to be upregulated in eEVs by 2.5-fold compared with sEVs, and increased expression has been shown to increase levels of brain-derived neurotrophic factor (BDNF) in a human breast cancer cell line.^75^ However, another study reported the opposite^76^ in a mouse model of stroke, although the reliability of both studies remains unclear. mmu-miR-126a-5p was upregulated in eEVs by 2.3-fold and has been found to be neurologically protective in a rat model of ischemic stroke, by reducing oxidative stress.^77^ It also seems to be involved in promoting blood-brain barrier (BBB) integrity, with higher expression of mmu-miR-126a-5p in microglia correlating with better BBB integrity and when deleted, causes BBB leakage in a mouse model of mass spectrometry (MS).^78^ Importantly, BBB leakage is an early sign of AD and is associated with cognitive decline.^79^ Finally, mmu-miR-142a-5p was upregulated in sEVs, and notably, increased expression of mmu-miR-142a-5p seems to be associated with AD, as inhibition of mmu-miR-142a-5p has been shown to prevent Aβ-induced loss of PSD-95.^80^ PSD-95 is involved in synaptic maturation and regulation of synaptic plasticity and is known to be reduced in patients with AD, leading to neuronal death and memory deficits.^80^ It is also involved in mitochondrial autophagy, with inhibition reducing damage to the hippocampus in an epileptic rat model by targeting PINK1.^81^ Proteomic analysis further revealed 929 proteins in exercise EVs, with significant differences in proteins associated with mitochondrial biogenesis and integrin signaling, pathways relevant to AD pathology. Activation of mitogen-activated protein kinases (MAPKs) were also enriched in eEVs compared with sEVs, which is consistent with findings showing that exercise activates MAPK in skeletal muscle, and activation of MAPKs, specifically MAP2K, has been implicated in neuronal regeneration in a model of spinal cord injury.^82^ Although some of these pathways may be involved in the benefits of exercise, mediated via EVs, may elicit in neurodegenerative diseases, some caution is warranted, as a large number of differentially expressed proteins within the eEV fraction were platelet-derived proteins, which led to significant enrichment in clotting pathways. This may be due to the large difference in concentration between protein cargo contained within EVs, which are highly heterogeneous and the co-isolated plasma proteins, including albumin and platelet-derived proteins, which are highly abundant even in effective EV isolations. This difference in expression level may mask the presence of proteins at lower concentrations and is highly sensitive to changes in concentration between samples, which can lead to significant differences in expression between conditions.^83^ However, it should be noted that EVs also play a role in platelet function and the enrichment of clotting pathways is consistent with other proteomic analysis of exercise released EVs.^54^^,^^84^ These findings underscore the complex role of EVs in mediating the systemic effects of exercise, suggesting that the differential cargo of EVs from physical activity could influence metabolic and neurodegenerative processes. This adds a new layer to our understanding of how exercise-induced changes in EV cargo could contribute to mitigating the metabolic dysfunction observed in AD, aligning with our findings that VET reduces REE to WT levels in APP/PS1Tg mice, independent of changes in food intake or activity level. Interestingly, mice treated with exercise-derived EVs displayed significantly higher blood glucose levels compared to those receiving sedentary EVs. A possible explanation for this observation is the documented increase in glycolytic enzymes within EVs released during exercise,^39^ which may enhance glycolysis in peripheral tissues such as the liver. This enhancement could improve the efficiency of glucose utilization and help normalize the abnormal energy expenditure phenotype observed in AD models like the APP/PS1Tg mice used in our study. Therefore, the elevated blood glucose levels may be reflective of increased glucose production and a reduced necessity for compensatory energy-wasting processes. It is important to acknowledge; however, that the transfer of eEV did not improve cognition in the APP/PS1Tg mice relative to the PBS or sEV groups in any of the behavioral tests assessed in this study. Together, our data demonstrate that increased energy expenditure may, indeed, contribute to the pathology of cognitive decline in AD, and that exercise training may improve cognitive decline, in part, by reducing elevated energy expenditure. Reduced energy expenditure cannot, however, be the sole mechanism by which exercise training mediates its benefit, because in the EV transfer experiment, a decrease in both TEE and, more importantly, REE, did not result in any cognitive improvements. Rather, our data suggest that the cognitive benefits of regular physical activity are either not mediated by EVs or only offer modest benefits that are not observed in the late stages of the disease.

In conclusion, we demonstrate that exercise training can normalize both aberrant energy expenditure and cognitive impairment in a well characterized mouse model of AD. Moreover, we demonstrate that exercise-released EVs potentially provide a mechanism through which exercise can cf. metabolic benefits in this mouse model, raising the possibility that EV transfer could be of potential therapeutic benefit to patients with AD in the future.

### Limitations of the study

Limitations of this study include only assessing behavioral changes without investigating molecular changes in the brain and the use of the APP/PS1 model of AD that only emulates Aβ pathology without neurofibrillary tangles.^41^^,^^42^ Moreover, the cognitive effects of weekly intranasal EV delivery under light anesthesia (2%–3% isoflurane) on the APP/PS1Tg remain unclear due to the absence of a no-sham control group. The weekly anesthesia and associated stress linked with delivery of EVs and PBS could have contributed to the substantial variation observed in the Barnes maze probe test. This might obscure any potential cognitive benefits provided by the treatment. Finally, while our approach utilized the highest dose of EVs (20 μg) per intranasal delivery, as recommended by previous research from Sterzenbach et al.,^57^ the frequency of once weekly delivery, intended to minimize anesthesia-related stress, may not have been enough to counteract the rapid and severe neurodegeneration characteristics of the APP/PS1Tg familial AD model.

Although findings in animal models of AD often show little effect in individuals with AD, some confidence can be gained from our data with respect to energy expenditure. Elevated REE is common in human patients with AD,^7^ and EVs isolated from exercising humans contain cargo involved in energy production,^39^ giving cause for optimism that these findings may translate to individuals with AD. Much more research is, however, required to demonstrate a link between EV transfer and cognition before pre-clinical EV transfer work can be translated to humans. While the difficulty of performing EV transfer experiments in human patients is significant, there is certainly a strong case for pursuing such research. Recently, Jia et al. demonstrated that a number of proteins, such as growth-associated protein 43 (GAP43), neurogranin, synaptosome-associated protein 25 (SNAP25), and synaptotagmin 1, were markedly reduced in EVs from patients with AD compared with controls.^85^ As EVs can be both engineered and delivered to the hippocampus via nasal delivery in mice,^57^ it is feasible that in the future, patients with AD could have their own EVs engineered to re-express these deficient proteins, which could then be delivered to the brain via the nasal route. However, it is important to consider whether the distribution of EVs, particularly to regions like the cortices, is sufficiently effective to influence cognition. This may be a limiting factor in the observed lack of cognition improvement in this work. Addressing this challenge is crucial for maximizing the therapeutic potential of EV-based treatments, ensuring that engineered EVs can indeed target and remediate the affected brain regions effectively.

## Resource availability

### Lead contact

Further information and requests for resources and reagents should be directed to and will be fulfilled by the lead contact, Mark A. Febbraio (mark.febbraio@monash.edu).

### Materials availability

This study did not generate new unique reagents.

### Data and code availability

• Proteomic data have been deposited at PRIDE as PXD050696 and are publicly available as of the date of publication. miRNA-seq data have been deposited at GEO as GSE262038 and are publicly available as of the date of publication.
• This paper does not report original code.
• Any additional information required to reanalyze the data reported in this paper is available from the lead contact upon request.

## Acknowledgments

The authors acknowledge the work of the staff at the animal research facilities at Monash Institute of Pharmaceutical Sciences, Monash University. The authors acknowledge the valuable contribution of Jason Howitt, who provided advice and guidance on the nasal delivery of EVs in mice. Additionally, the authors are grateful for the guidance of Simon McKenzie-Nickson and Arthur Christopoulos in the design of the behavioral assay protocols.

M.A.F. is a Senior Principal Research Fellow of the 10.13039/501100000925NHMRC (APP1116936) and is also supported by an NHMRC Investigator Grant (APP1194141). This work was also supported by an ARC Discovery Project Grant awarded to M.A.F and G.A.R. (DP220102500).

## Author contributions

Conceptualization: M.A.F. and M.W.; methodology: O.K.F., M.W. and M.A.F.; analysis: O.K.F. and M.W.; bioinformatics: O.K.F. and M.W.; resources: Monash Animal Research Platform (MARP); writing – original draft: O.K.F. and M.A.F.; writing – review and editing: all authors; visualization: O.K.F., C.L.E., and M.d.M.; supervision: M.A.F. and M.W.; project administration: O.K.F.; funding acquisition: M.A.F. and G.A.R.

## Declaration of interests

M.A.F. is a shareholder and scientific advisor for N-Gene Pharmaceuticals. M.A.F. is the founder and shareholder of Celesta Therapeutics.

## STAR★Methods

### Key resources table

**Table:** 

REAGENT or RESOURCE	SOURCE	IDENTIFIER
**Antibodies**		

Iba1/AIF-1 (E4O4W) XP® Rabbit mAb	Cell Signaling Technology	Cat# 17198; RRID:AB_2820254
TNF-alpha Ab	Cell Signaling Technology	Cat# 3707; RRID:AB_2240625
β-Actin Ab	Cell Signaling Technology	Cat# 4967; RRID:AB_330288
IL-1beta (11E5) Ab	Santa Cruz Biotechnology	Cat# sc-52012; RRID:AB_629741
Albumin Ab	Cell Signaling Technology	Cat# 4929; RRID:AB_2225785
Alix (3A9) Mouse mAb	Cell Signaling Technology	Cat# 2171; RRID:AB_2299455
ApoA1 Polyclonal Ab	Thermo Fisher Scientific	Cat# PA5-29557; RRID:AB_2547033
ApoB Ab	Abcam	Cat# ab20737; RRID:AB_2056954
Rabbit Anti-CD9 Ab, Unconjugated	Sigma-Aldrich	Cat# C999; RRID:AB_2076045
Anti-CD81 (D5O2Q) Rabbit mAb	Cell Signaling Technology	Cat# 10037; RRID:AB_2714207
Grp94 Ab	Cell Signaling Technology	Cat# 2104; RRID:AB_823506
HSP70 Ab	Cell Signaling Technology	Cat# 4872; RRID:AB_2279841
Caveolin-1 Ab	Cell Signaling Technology	Cat# 3238; RRID:AB_2072166
Anti-rabbit IgG, HRP-linked Ab	Cell Signaling Technology	Cat# 7074; RRID:AB_2099233
1E8-4b non-commercial	Tammer et al.^86^	N/A
Anti-mouse IgG, HRP-linked	Cell Signaling Technology	Cat# 7076; RRID:AB_330924

**Chemicals, peptides, and recombinant proteins**		

ImmPACT® DAB Substrate Kit, Peroxidase (HRP)	Vector Labs	SK-4105
Gill′s Hematoxylin Solution, No. 2	Santa Cruz Biotechnology	Cat# sc-24973

**Critical commercial assays**		

Pierce™ Dilution-Free™ Rapid Gold BCA Protein Assay	Thermo Fisher Scientific	Cat# A55860
NEFA C-test kit	Wako	Cat# 279-75401
Mouse Ultrasensitive Insulin ELISA kit	Alpco Diagnostics	Cat# 80-INSMSU-E01; RRID:AB_2792981
Amyloid beta 1-42 ELISA	Thermo Fisher Scientific	Cat# KHB3441
Mouse IL-10 ELISA Kit	R&D Systems	Cat# M1000B
Mouse IL-1 beta/IL-1F2 ELISA Kit	R&D Systems	Cat# MLB00C
Mouse Corticosterone ELISA Kit	Crystal Chem	Cat# 80556

**Deposited data**		

Proteomic data	PRIDE	PXD050696
miRNA-seq data	Gene Expression Omnibus (GEO)	GSE262038

**Experimental models: Organisms/strains**		

Mouse: B6C3-Tg(APPswe,PSEN1dE9)85Dbo/Mmjax	MMRRC	Cat# 034829-JAX; RRID:MMRRC_034829-JAX
Mouse: C57Bl/6J	Monash Animal Research Platform	N/A

**Software and algorithms**		

GraphPad Prism 9.2.0	Graphpad	RRID:SCR_002798
R package version 4.10.2	r-project free software	RRID:SCR_001905
Fiji (ImageJ) v2.14.1	Open Source NIH software	N/A
ShinyGO v0.81	South Dakota State University	RRID:SCR_019213
Spectronaut™	Biognosys	N/A
ezTrack	Pennington et al.^87^	N/A

**Other**		

Automatic Fraction Collector (AFC)	IZON Science	AFC-V1
qEV2	IZON Science	IC2-35
qEV10	IZON Science	IC10-35
Ultra-15 Centrifugal Filter	Merck	UFC910008
CDS Empore™ C18 Extraction Disks	Thermo Fisher Scientific	13-110-019
S-Trap™ Micro Columns	Protifi	C02-micro

### Experimental model and study participant details

Animal experiments were approved by the institutional animal ethics committee (Monash University Animal Ethics Committee), and animals were maintained in accordance with the Australian code of practice criteria for the care and use of animals for scientific purposes. APP/PS1Tg (originally obtained from The Jackson Laboratory, ME, USA, JAX MMRRC Stock# 034829, expressing a chimeric mouse/human amyloid precursor protein (Mo/HuAPP^695swe^) and mutant human presenilin 1 (PS1^Δexon9^)^41^^,^^42^) and wild-type non-transgenic mice were used to establish in-house colonies. Sixty male WT littermates and forty-six male APP/PS1Tg mice were randomly assigned to different treatment groups at 6 wk of age. Male mice were chosen to minimize to avoid potential variations linked to the reproductive cycles of females. Mice were group-housed under standard conditions (12-hour light/dark cycle; temperature, 22°C ± 2°C; humidity, 55% ± 5%; *ad libitum* access to standard chow and water). At 6.5 months of age, the voluntary running groups were dual-housed with two running wheels (locked for sedentary and unlocked for exercise), and the wheel distance was monitored continuously. Mice had access to the wheels for an additional 6 months. Body composition was assessed every two months using magnetic resonance imaging (echoMRI™, TX, USA).

### Method details

#### Metabolic phenotyping

At 11-months of age, mice were metabolically phenotyped using the Promethion® metabolic cage system. Parameters measured included oxygen uptake (VO2) and carbon dioxide output (VCO2), continuously tracked to determine the respiratory exchange ratio (RER) and calculate energy consumption (EE). Infrared beams were used to gauge activity by recording beam interruptions, and sleep cycles were deduced from extended periods of no interruptions, suggesting inactivity. Food consumption was also monitored continuously. These parameters were aggregated on an hourly basis across a 48-hour period, with totals for the entire period as well as separate totals for light and dark phases derived from summing these hourly data. Due to significant fluctuations in food consumption measurements, which arise when mice displace food from the hopper, any hourly consumption exceeding 2 grams (g) was adjusted to 0.8g. To reduce stress and promote consistent behavior, mice were acclimated to individual enclosures for 3 days before the experiments. Subsequently, they were transferred to Promethion® metabolic cages for the duration of the 48-hour measurement period. Mice were returned to their home cages (dual-housed for voluntary running groups, 3-5 mice for EV treatment groups). Plasma glucose levels were measured using an Accu-Chek glucometer with compatible glucose test strips. Plasma non-esterified fatty acid (NEFA) concentrations were determined using the FUJIFILM Wako NEFA-C assay kit (FUJIFILM Wako Chemicals, JP, 279-75401) according to the manufacturer's instructions. Plasma insulin levels were assessed using the Mouse Ultrasensitive Insulin ELISA kit (ALPCO Diagnostics, NH, USA, 80-INSMSU-E01), according to the manufacturer's instructions.

#### Rotarod

Commencing at 4.5 months of age, the mice underwent the rotarod test every 2 months until the study endpoint. Before the first test, the mice were acclimatized to the rotarod test one day before the test. This was done by having each mouse undergo two 2 min sessions on the rotarod set at a speed of 4 revolutions per minute (RPM), followed by a 2 min session with the speed slowly increasing to a maximum of 40 RPM. The following day, the first trial was carried out, with each mouse undergoing three five-minute sessions with the rotarod speed slowly increasing to 40 RPM. The time spent on the rotarod for each run was measured and averaged over three runs to determine the time spent on the rotarod for each mouse. For subsequent trials, no acclimatization tests were conducted.

#### Locomotor Activity Test (LMA)

One week after the rotarod test, mice underwent the LMA test, every 2 months until the study endpoint. Briefly, the mice were placed in a behavioral testing room for 1 h in their home cage with free access to food and water. They were then placed in a white box (38cm x 38cm x 38cm), and their activity was recorded using an overhead video camera for 90 min. Their movements were tracked using Viewer 3 software (Biobserve, Bonn, Germany).

#### Spontaneous alternation Y-Maze

Mice underwent the spontaneous alternation Y-maze three times, at 4.7 months, 8.5 months and 11.5 months of age. Mice were removed from their home cage and placed in one of the arms of the Y-maze (the starting arm was chosen randomly for each mouse). Cues with black and white shapes were placed at the end of each arm. Fresh wood shavings were placed on the floor of the Y-maze. An overhead video camera was used to track the movements. White overhead lights were set to dim. Tracking software (Viewer 3, Biobserve, Bonn, Germany) was activated immediately after the mouse was placed in the maze. Arm entry was defined as the entry of the head and shoulders of the mouse across the threshold of the central zone and into the arm, and the snout of the animal was oriented toward the end of the arm. Spontaneous alternation was defined as a sequential entry into the three arms. Spontaneous alternation behavior was recorded for 10 min.

#### Barnes Maze

Mice were habituated on day one by placing them on the maze for 5 min and in the safe house for 1 min (location opposite to that used in the acquisition trial). The safe house was attached for the acquisition trials at a different location from that used for the habitation trial (different locations for each mouse, not directly in line with any of the cues to reduce saliency). The position of the safe house then remained at this fixed location relative to the spatial cues in the room (four 2D colored shapes on each of the walls and two 3D cues in the corners of the room, all equal distances from the Barnes maze for the duration of the training period). The training consisted of four acquisition trials/d (3 min limit per trial). Bright light above the maze acted as an adverse stimulus. These were switched on after the mouse was released from the bucket. The trial concluded once the mouse entered the safe house, or 3 min had elapsed. Three days after the final acquisition training session, the mice underwent a 90 s probe trial in which the safe house was removed from the apparatus. The probe trial was administered in a manner similar to the acquisition trials, with a time limit of 90 s. The errors to the target hole were calculated for each mouse. Heat maps were generated using ezTrack.^87^

#### EV donor mice

Eight-week-old C57BL/6 mice were assigned to two groups: exercise and sedentary. In the exercise group, the mice were acclimatized for three days on a treadmill (day 1:10 m/min. day 2: 12 m/min. day 3: 14 m/min). Three days later, they underwent a 60-minute bout to exhaustion, increasing in speed from 10 m/min to 24m/min by 2m/min every 10 min. Exhaustion was defined as the inability to continue at the selected running speed despite gentle encouragement with a brush for 5-10s). Once exhaustion was reached, the mice were subjected to 4% isoflurane until they were unconscious before cardiac puncture was performed to collect blood.

#### EV isolation

After the mice were determined to be fatigued, blood was collected via cardiac puncture and centrifuged at 2500 × g for 10 min at room temperature (RT). The supernatant was transferred to a new tube and centrifuged again at 2500 × g for 15 min at RT before being transferred to a new tube and frozen at -80°C. This was to ensure minimal contamination from platelet derived EVs ex-vivo 52. Isolation via SEC was performed with the IZON Science Automatic Fraction Collector and qEV2 and qEV10 columns according to the manufacturer’s instructions (IZON Science, NZ). After the initial characterization using the qEV2 column, further isolation was performed using the qEV10 column. After isolation, the EV-rich (1-3) and non-EV fractions (5-7) were pooled and concentrated using Amicon Ultra-15 Centrifugal Filter Units (4000 g spin for 50 mins) down to 500 μl. EV concentration was quantified using a Qubit fluorometer (Thermo Fisher Scientific, MA, USA).

#### Nanoparticle Tracking Analysis (NTA)

NTA was performed on the pooled fractions using the ZetaView® x30 system (Particle Metrix, Germany) with a laser wavelength of 488 nm and data were collected from 11 positions; all other parameters were set to their default values.

#### Transmission Electron Microscopy (TEM)

EV samples at a concentration of 0.2 mg/mL were analyzed using a JEOL JEM-2010 transmission electron microscope operating at 200 kV. To prepare the samples, carbon-layered copper grids with a mesh size of 400 μm were used, and the grids were fixed for 2 min. Any excess sample was removed by blotting, and the samples were subjected to two rounds of negative staining with 10 μL of 2% w/v uranyl acetate solution (Electron Microscopy Services, PA, USA).

#### Western blot analysis

For markers of neuroinflammation, total protein was extracted from fresh-frozen right hemispheres and for markers of EVs and potential plasma protein contaminants, EVs were isolated via SEC from exercise and sedentary plasma using a 10 ml qEV10 35 nm column (IZON Science, NZ). Fractions (7 × 5 ml) were collected and concentrated using Amicon Ultra-4 Centrifugal Filter Units (4000 × g spin for 20 min) or pooled into EV-rich (1-3) and non-EV fractions (5-7) and then concentrated using Amicon Ultra-15 Centrifugal Filter Units (4000 g spin for 50 min). Protein concentration was quantified using a Pierce BCA protein assay kit (Thermo Fisher Scientific, MA, USA). 10μg of protein was diluted in Laemmli buffer and loaded into each well. Proteins were separated using 10-12% SDS-PAGE on polyacrylamide gels, transferred to membranes (Trans-Blot Turbo System, 7-minute protocol, Bio-Rad, CA, USA), and blocked with 2.5% bovine serum albumin (BSA). After addition of primary antibodies, membranes were incubated overnight, followed by 60 min incubation with secondary antibodies and detection using Pierce ECL/Super ECL detection reagent (Thermo Fisher Scientific, MA, USA) on the ChemiDoc Gel Imaging System (Bio-Rad, CA, USA). Primary antibodies used were IBA1 (17198, Cell Signaling, MA, USA. 1:1000), TNFα (3707S, Cell Signaling, MA, USA. 1:1000), β-actin (4967, Cell Signaling, MA, USA. 1:1000), IL-1β (SC-52012, Santa Cruz Biotechnology, TX, USA. 1:750), Albumin (4929, Cell Signaling, MA, USA. 1:1000), ALIX (3A9) (2171, Cell Signaling, MA, USA. 1:1000), ApoA (PA5-29557, Thermo Fisher Scientific, MA, USA. 1:1000), ApoB (ab20737, ABCAM, UK. 1:1000), CD9 (C9993, Sigma-Aldrich, MO, USA. 1:1000), CD81 (10037, Cell Signaling, MA, USA. 1:1000), Grp94 (2104, Cell Signaling, MA, USA. 1:1000), HSP70 (4872, Cell Signaling, MA, USA. 1:1000) and Caveolin-1 (3238, Cell Signaling, MA, USA. 1:1000). The secondary antibody used was goat anti-rabbit (7074, Cell Signaling, MA, USA. 1:3000 with IBA1, TNFα, β-actin and IL-1β. 1:1000 for all other primary ABs).

#### Immunohistochemistry

Hemisected brains were stored at 4°C in 30% sucrose solution. The brains were removed from sucrose, and the olfactory bulb and cerebellum were excised at room temperature using a scalpel. The remaining brain tissue was embedded in Tissue-Tek O.C.T. compound (Sakura Finetek, JPN, 4583) and then frozen with methylbutane in liquid nitrogen. The frozen brains were stored at –80°C until sectioning. Brain tissues were sectioned at a thickness of 10 μm using a cryostat set at –16°C. Sections were mounted onto slides and allowed to air dry for 20–60 minutes before being stored at –80°C until staining. Prior to staining, slides were allowed to reach room temperature (∼40 minutes on bench). Sections were washed three times for 5 minutes each in PbTx + Tween solution (phosphate-buffered saline (PBS) containing 0.2% Triton X-100 and 0.1% Tween 80). Blocking was performed with 5% BSA in PbTx + Tween for 1 hour at room temperature. After a 5-minute wash in PBS, sections were incubated with the primary antibody (1E8-4b non-commercial^86^^,^^88^) diluted 1:2000 in blocking buffer. Incubation was carried out in the dark at room temperature for 1.5 hours (150 μL per section). Sections were then washed three times for 5 minutes each in PbTx + Tween. Secondary antibody incubation was performed using anti-mouse IgG HRP-linked antibody (7076, Cell Signaling, MA, USA) diluted 1:2000 in PbTx + Tween for 1 hour at room temperature. Sections were washed three times for 5 minutes each in PbTx + Tween. The diaminobenzidine (DAB) substrate was prepared according to the manufacturer's instructions for the ImmPACT DAB Kit, peroxidase (Vector Laboratories, CA, USA, SK4105). DAB solution was applied to each section for 5 minutes. Counterstaining was performed by immersing the sections in Gill's hematoxylin solution No. 2 (Santa Cruz Biotechnology, TX, USA, Sc24973) for 30 seconds, followed by rinsing. Sections were dehydrated sequentially in the fume hood by immersing twice in water, followed by immersions in 100% ethanol, 90% ethanol, 70% ethanol, and finally two rinses in xylene. Slides were then mounted with DPX mounting medium (Sigma-Aldrich, MO, USA, 06522) and imaged using a Leica DMi8 widefield microscope (Leica Microsystems, DE). The entire left hemisphere was captured. In Fiji, a grid overlay of 255,000 μm^2^ was applied to each section. Two blinded scorers independently counted each DAB-positive stain over the entire section, and the counts were averaged for each section.

#### Cytokine, corticosterone/cortisol and human amyloid-β 42 ELISA

The levels of human amyloid-β42 were assessed in the hippocampus and cortex of APP/PS1Tg mice using ELISA (Thermo Fisher Scientific, MA, USA, KHB3441). Briefly, hippocampal and cortical tissues were diluted 10-fold of their mass with 50 mM Tris-HCl buffer (pH 8.0) containing cOmplete™ mini protease inhibitor and phosSTOP™ phosphatase inhibitor cocktail tablets to prevent the proteolysis of Aβ peptides. To extract parenchymal Aβ, the tissue from each mouse brain cortex was subjected to brief homogenization for 10 seconds per sample, utilizing a tissue homogenizer and then further sonicated at a frequency of 80% amplitude for 1 min (1 s on and 1 s off) at 4°C. After homogenization, the mixture was centrifuged at 18,000 x g for 30 minutes at a temperature of 4°C. The clear layer (supernatant) obtained post-centrifugation was then diluted further by a factor of 100 using standard diluent buffers. This step was based on preliminary experiments which showed that such dilution yields absorbance values that align with the standard curve established by the provided standards. Levels of amyloid-β42 within the hippocampus and cortices were normalized to total protein. The concentrations of IL-10, IL-1β, and corticosterone/cortisol present in the plasma, EV, and EV-poor fractions were assessed using ELISA. IL-10 (Quantikine Mouse IL-10, M1000B) and IL-1β (Quantikine Mouse IL-1 beta/IL-1F2, MLB00C), (both from R&D Systems, MN, USA). Corticosterone/cortisol (Mouse Corticosterone, ELISA kit 80556; Crystal Chem, IL, USA). All experiments were performed according to the manufacturer’s specifications.

#### miRNA sequencing of mouse EVs

EVs were isolated using qEV10 columns from ∼10 ml of exercised and sedentary mouse plasma. EV-rich fractions (1-3) were concentrated using Amicon Ultra-15 Centrifugal Filters to a volume of 600 μL before RNA was extracted using the qEV RNA Extraction Kit according to the manufacturer’s instructions (IZON Science, NZ). Detection, quality assessment, and quantification of miRNAs were performed using an Agilent Bioanalyzer 2100 (Agilent Technologies, CA, USA). The samples were analyzed for total RNA using an Agilent RNA 6000 Pico kit (Agilent Technologies, CA, USA). Small RNA NGS libraries were prepared using Illumina’s TruSeq Small RNA Library Prep kit (Illumina Inc., CA, USA) and were single end sequenced at a read length of 50 nucleotides (nt). Following the manufacturer's instructions, sequencing was conducted on an Illumina HiSeq2000 platform (Illumina Inc., CA, USA) using the HiSeq SBS Kit V4 (Illumina Inc., CA, USA). Endogenous reference miRNAs within the NGS dataset were determined using the NormFinder algorithm. Counts for individual miRNA species were normalized to the total number of miRNA counts and fold change between exercise EV and sedentary EVs. Since only single replicates containing RNA extracted from 20 mice were sequenced for exercise and sedentary conditions, no significance could be calculated between species.

#### Proteomic analysis of mouse EVs

EVs were isolated using ultracentrifugation (2x 2 h 100,000 × g spins) from two acutely exercised and two sedentary mice. 50ul of S-Trap™ (ProtiFi, NY, USA) lysis buffer was added to the pellets before incubation at 70°C for 10 min with agitation at 1500 rpm, which was followed by sonication for 15 cycles (30 s on and 30 s off, 4°C) at a frequency of 30% amplitude. The debris was removed by centrifugation at 10,000 × g for 5 min and the supernatant was transferred to a new tube. The samples were reduced, alkylated, and digested overnight (37°C for 18 h) according to the S-Trap™ protocol (ProtiFi, NY, USA). After the peptides were eluted, ACN was evaporated until approximately 100 μL remained. The pH was measured and adjusted to between 2-4 using Formic Acid (FA). In house stage-tips were created using 2 layers of C18 membrane (CDS Empore™, Thermo Fisher Scientific, MA, USA) in 200 μL pipette tips. The stage tips were activated with 40 μL methanol, followed by centrifugation at 1200 × g for 1 min. The membrane was equilibrated using 40 μL of 0.1% formic acid before centrifugation at 1200 × g for 1 min. The samples were loaded before spinning at 800 × g for 5 min. To ensure purity, the sample was washed four times with 40 μL of 0.1% formic acid, with each wash accompanied by a minute centrifugation at 1200 × g. For elution, a mixture of 2 × 50 μL 70% ACN with 0.1% formic acid was used, and the mixture was centrifuged at 500 × g for 5 min. The samples were dried and resuspended with iRT, ACN, and formic acid before loading onto Acclaim PepMap RSLC C18 columns (Thermo Fisher Scientific, MA, USA) using a Dionex UltiMate™ 3000 RSLCnano Nano LC system (Thermo Fisher Scientific, MA, USA). MS data were acquired on an Orbitrap Q Exactive HF (Thermo Fisher Scientific, MA, USA) operated in data-independent mode (DIA). Following the methodology outlined in,^89^ we used 25 fixed 14 m/z windows covering a range of 382–970 Da. Fragmentation was performed at a resolution of 17,500, (automatic gain control target of 2e5, auto-adjusted maximum injection time, and normalized collision energy of 27.0). The MS data were searched using Spectronaut™ (version 17.0, Biognosys, CH) against *Mus musculus* (mouse) -10090_validated (downloaded on 28-02-2022) UniProt FASTA database. Mass tolerances were determined by its extensive mass calibration, using a correction factor of 1. At both precursor and fragment levels, the most intense data-point within the m/z tolerance was selected. Peptide identification was based on the default values with a 1% Q-value cutoff for precursors and proteins. Dynamic iRT was employed for retention time prediction. Interference correction was applied at the MS2 level for accurate quantification, which also included cross-run normalization based on total peak areas, with the significance threshold set to 0.01.^89^ Pathway enrichment analysis was performed using ShinyGO^90^ with FDR cutoff set to 0.05, KEGG pathway dataset^91^ and minimum pathway size set to 5.

#### EV transfer

Exercise and sedentary EVs at a dose 20 μg of along with PBS were delivered weekly via nasal delivery starting at 4.5 months old. Before delivery, mice were lightly sedated via isoflurane inhalation.

### Quantification and statistical analysis

One-way, two-way, and mixed model ANOVA were performed using GraphPad Prism version 9.2.0 for Mac (GraphPad Software, California, USA) for the general statistical analysis. For the proteomic analysis, differences between sedentary and exercise EVs were assessed using student t-test with significance determined by p<0.05 and FC >1.5. Negative binomial regression was performed using the R software (R Foundation for Statistical Computing, Austria) for count data analysis. Specific comparisons post-ANOVA were facilitated by Tukey’s post hoc test.
