# Supplementary material for: Extracellular vesicles contribute to the beneficial effects of exercise training in APP/PS1 mice
Source: iScience. 2025 Jan 4;28(2):111752. doi: 10.1016/j.isci.2025.111752 (PMC11787611; doi:10.1016/j.isci.2025.111752)
Supplement: Document S1. Figures S1–S3 [file mmc1.pdf]

iScience, Volume *28*

Supplemental information

Extracellular vesicles contribute to the beneficial effects of exercise training in APP/PS1 mice

Oliver K. Fuller, Emma D. McLennan, Casey L. Egan, Nimna Perera, Lauren V. Terry, Jae Pyun, Mariana de Mendonca, Guilherme Defante Telles, Benoit Smeuninx, Emma L. Burrows, Ghizal Siddiqui, Darren J. Creek, John W. Scott, Michael A. Pearen, Pamali Fonseka, Joseph A. Nicolazzo, Suresh Mathivanan, Anthony J. Hannan, Grant A. Ramm, Martin Whitham, and Mark A. Febbraio

FIGURE S1

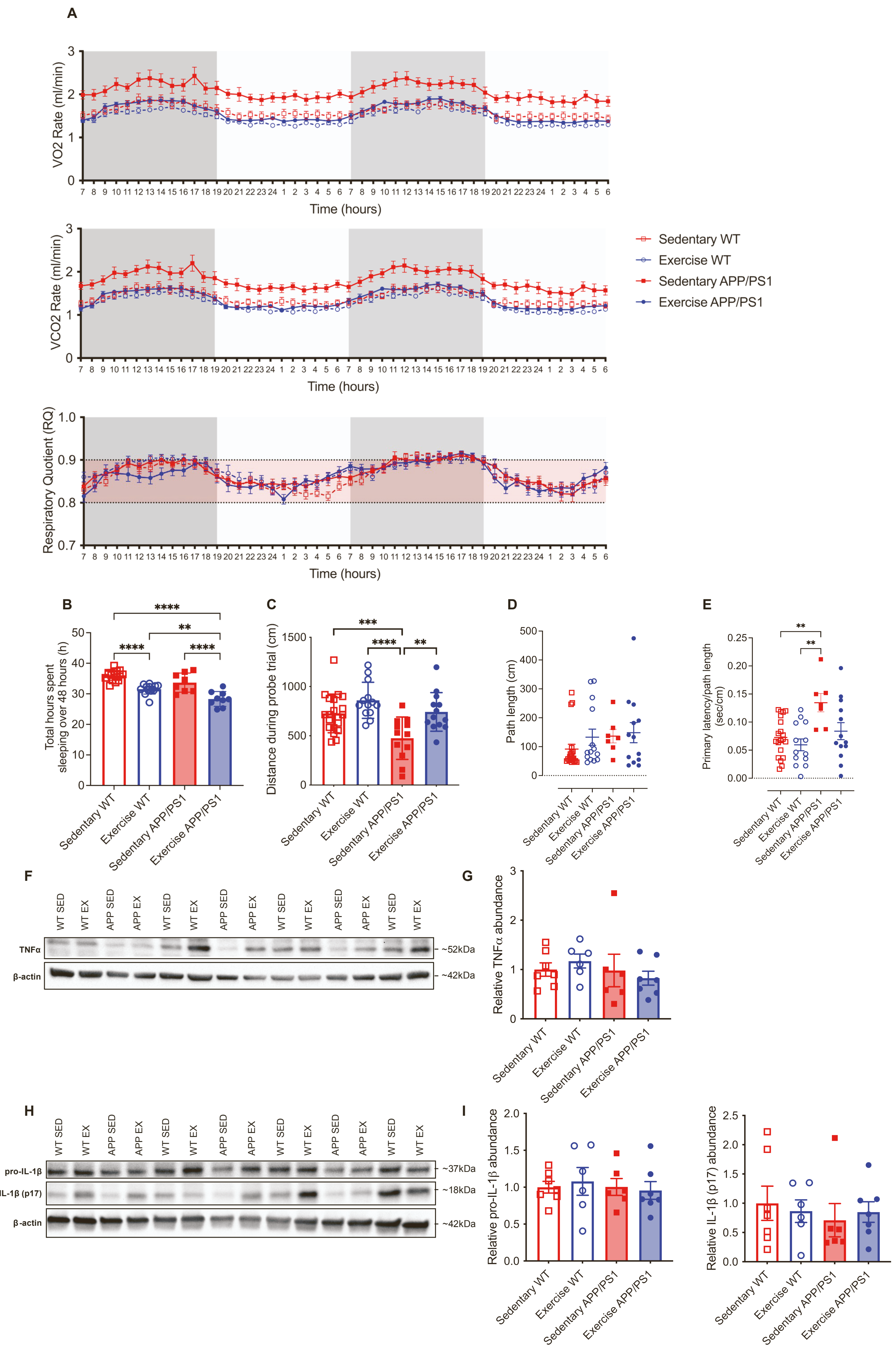

A
VO2 Rate (ml/min)
VCO2 Rate (ml/min)
Respiratory Quotient (RQ)
Time (hours)
Sedentary WT
Exercise WT
Sedentary APP/PS1
Exercise APP/PS1
B
Total hours spent sleeping over 48 hours (h)
C
Distance during probe trial (cm)
D
Path length (cm)
E
Primary latency/path length (sec/cm)
F
WT SED
WT EX
APP SED
APP EX
TNFα
β-actin
~52kDa
~42kDa
G
Relative TNFα abundance
H
pro-IL-1β
IL-1β (p17)
β-actin
~37kDa
~18kDa
~42kDa
I
Relative pro-IL-1β abundance
Relative IL-1β (p17) abundance

Figure S1: Voluntary exercise training restores metabolic parameters, reduces sleep time, and improves spatial exploration in male APP/PS1Tg mice without altering inflammatory markers. A Oxygen consumption (top), carbon dioxide production (middle), and respiratory quotient (RQ) (bottom) for groups with access to wheels; **B** Time spent sleeping over 48 h during metabolic phenotyping; **C** Distance travelled during the probe trial for each group of exercised and sedentary mice; **D** Path length (distance from start box to target hole) during probe trial for each group of exercised and sedentary mice; **E** Primary latency normalized to path length for each group. Three sedentary APP/PS1 did not find target hole during probe trial and so were excluded; **F** Representative Western blot images showing TNFa protein expression in brain tissue samples from Sedentary and Exercise WT and APP/PS1 groups. β-actin loading control shown below; **G** Quantification of TNFα protein levels normalized to β-actin and expressed relative to the sedentary WT group; **H** Representative Western blot images showing pro-IL-1β and IL-1β (p17) protein expression in brain tissue samples from Sedentary and Exercise WT and APP/PS1 groups. β-actin loading control shown below; **I** Quantification of pro-IL-1β and IL-1β (p17) protein levels normalized to β-actin and expressed relative to the sedentary WT group. Significance was calculated using two-way/mixed model ANOVA Tukey post hoc $^{**}P<0.01$, $^{***}P<0.001$, $^{****}P<0.0001$. Sedentary WT n=17-21, exercise WT n=12-13, sedentary APP/PS1 n=7-12, exercise APP/PS1 n=9-13. All data are presented as the group mean ± SEM.

FIGURE S2

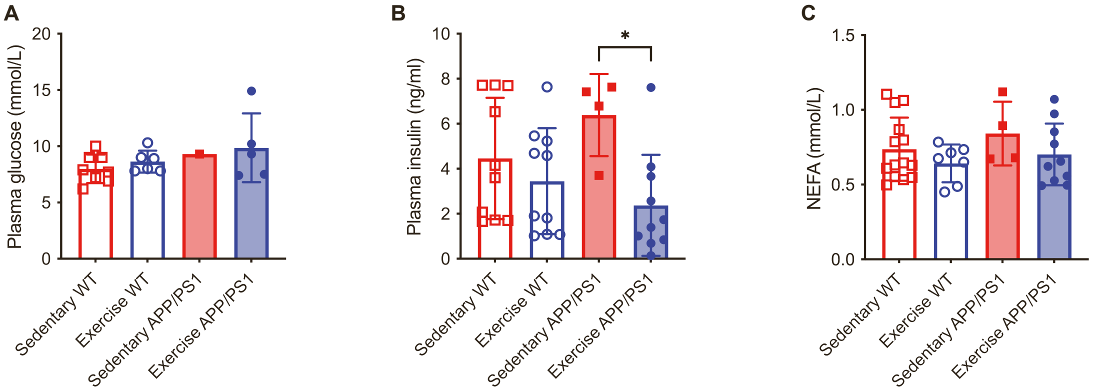

Figure S2: Voluntary exercise training reduces blood insulin levels in male APP/PS1Tg mice without affecting glucose or NEFAs. A Plasma glucose; **B** Insulin and **C** NEFAs for Sedentary and Exercise WT and APP/PS1 mice. Significance was calculated using two-way/mixed model ANOVA Tukey post hoc *P<0.05. Sedentary WT n=7-14, exercise WT n=6-10, sedentary APP/PS1 n=1-4, exercise APP/PS1 n=5-10. All data are presented as the group mean ± SEM.

FIGURE S3

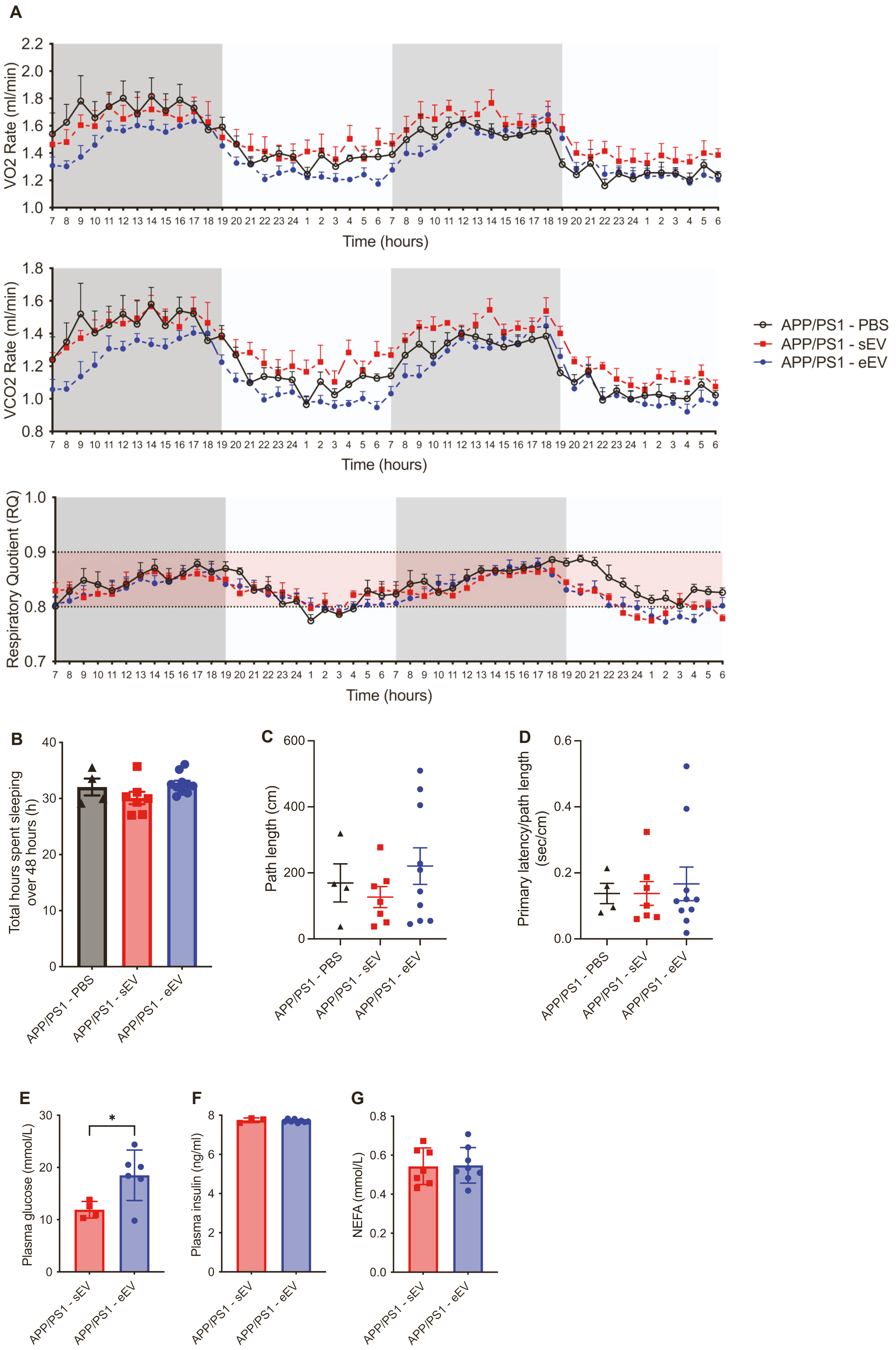

Figure S3: Intranasal extracellular vesicle delivery does not alter metabolic or behavioral parameters but elevates blood glucose levels in APP/PS1Tg mice treated with exercise-released EVs. A Oxygen consumption (top), carbon dioxide production (middle), and respiratory quotient (RQ) (bottom) for the groups treated with PBS and EVs; **B** Time spent sleeping for over 48 h during metabolic phenotyping; **C** Path length (distance from start box to target hole) during probe trial for the groups treated with PBS and EVs; **D** Primary latency normalized to path length for each group; **E** Plasma glucose; **F** Insulin and **G** NEFAs for APP/PS1 mice treated with sedentary and exercise EVs. Significance was calculated using one-way/mixed model ANOVA Tukey post hoc *P<0.05, **P<0.01, ***P<0.001, ****P<0.0001. APP/PS1 PBS, n=4; APP/PS1 sEV, n=3-7; APP/PS1 eEV, n=6-10. All data are presented as the group mean ± SEM.
